# Medical Care and Payment for Diabetes in China: Enormous Threat and Great Opportunity

**DOI:** 10.1371/journal.pone.0039513

**Published:** 2012-09-26

**Authors:** Wenying Yang, Wenhui Zhao, Jianzhong Xiao, Rui Li, Ping Zhang, Katarzyna Kissimova-Skarbek, Erin Schneider, Weiping Jia, Linong Ji, Xiaohui Guo, Zhongyan Shan, Jie Liu, Haoming Tian, Li Chen, Zhiguang Zhou, Qiuhe Ji, Jiapu Ge, Gang Chen, Jonathan Brown

**Affiliations:** 1 Chinese-Japanese Friendship Hospital, Beijing, People's Republic of China; 2 Graduate School, Peking Union Medical College and Chinese Academy of Medical Sciences, Beijing, People's Republic of China; 3 Centers for Disease Control and Prevention, Atlanta, Georgia, United States of America; 4 Institute of Public Health, Jagiellonian University Medical College, Krakow, Poland; 5 International Diabetes Federation, Brussels, Belgium; 6 National Bureau of Asian Research, Seattle, Washington, United States of America; 7 Shanghai Jiaotong University Affiliated Sixth People's Hospital, Shanghai, People's Republic of China; 8 Peking University People's Hospital, Beijing, People's Republic of China; 9 Peking University First Hospital, Beijing, People's Republic of China; 10 First Affiliated Hospital, Chinese Medical University, Shenyang, Liaoling, People's Republic of China; 11 Shanxi Province People's Hospital, Taiyuan, Shaanxi, People's Republic of China; 12 West China Hospital, Sichuan University, Chengdu, Sichuan, People's Republic of China; 13 Qilu Hospital of Shandong University, Jinan, Shandong, People's Republic of China; 14 Xiangya Second Hospital, Changsha, Hunan, People's Republic of China; 15 Xijing Hospital, Fourth Military Medical University, Xi'an, Shaanxi, People's Republic of China; 16 Xinjiang Uygur Autonomous Region's Hospital, Urmqi, Xinjiang, People's Republic of China; 17 Fujian Provincial Hospital, Fuzhou, Fujiang, People's Republic of China; University of Milan, Italy

## Abstract

**Background:**

The Diabetes Impact Study followed up a large national population-based screening study to estimate the use of and expenditures for medical care caused by diabetes in China and to ascertain the use and cost of essential basic medicines and care.

**Methods:**

In 2009–10, the study team interviewed 1482 adults with diabetes and 1553 adults with glucose tolerance in the normal range from population-based random samples at 12 sites in China. The response rate was 67%.

**Findings:**

After adjusting for age, sex, and urban/rural location, people with diabetes received 1.93 times more days of inpatient treatment, 2.40 times more outpatient visits, and 3.35 times more medications than people with normal glucose tolerance (all p<0.05). Adjusted expenditures for medical care were 3.38 times higher among people with diabetes than among people with normal glucose tolerance (p<0.01, unadjusted 3.97). Persons who were diagnosed with ≥10 years prior to the survey paid 3.75 times as much for medical care as those with ≤5 years of diagnosed diabetes. Among persons with diabetes, 45.2% took medication to control blood sugar, 21.1% took an antihypertensive medicine, 22.4% took daily aspirin, and 1.8% took a statin. Over the three months before the interview, 46.1% of persons with diabetes recalled seeing a doctor, 48.9% recalled a blood pressure measurement, and 54.5% recalled a blood sugar test. Over the year preceding the interview, 32.1% recalled a retinal screening and 17.9% recalled a foot examination.

**Conclusions:**

In China, health care use and costs were dramatically higher for people with diabetes than for people with normal glucose tolerance and, in relative terms, much higher than in industrialized countries. Low-cost generic medicines that would reduce diabetes expenditures were not fully used.

## Introduction

Noncommunicable diseases (NCDs) account for the majority of disability and premature death in nearly all of the world's countries [1]. Diabetes mellitus (DM) is an NCD of particular interest because untreated DM can lead to a variety of disabling, life-threatening, and expensive complications, including stroke, heart attack, renal disease, neuropathy, peripheral artery disease, lower-limb amputation, and visual impairment. In 2011, DM was associated with 4.6 million deaths worldwide and consumed at least 465 billion current U.S. dollars (USD) in health care resources [2], [3]. In fact, DM causes more deaths annually than HIV and malaria combined [3]. In most of the world, type 2 diabetes, the predominant form, occurs in people on average ten years sooner and at a lower body mass index (BMI) than in populations of European heritage, [4]–[6] and is linked to history of famine as well as to current diet and lack of physical activity [7], [8]. Three-quarters of persons with diabetes live in low- and middle-income countries (LMICs) [3].

In LMICs, the impact of DM falls both on individuals and their families: disability or death from DM can lead to family poverty from loss of income and from the expense of medical care, and then to malnutrition, interruption of education, and the loss of a business or a farm [9]. When diabetes prevalence is high, impoverishment at the family level will cumulate to economic stagnation and social instability, which harm entire communities and retards economic and social development nationally [9].

Information on the availability, cost, and quality of medical care for DM is generally not available for LMICs. Documenting access to care is particularly important because complications from DM, which can be devastating, could largely be prevented by wider use of inexpensive generic medicines, such as metformin, sulphonylureas, statins, angiotensin-converting-enzyme (ACE)-inhibitors, and other classes of blood pressure-lowering medicines [10]–[13]. Because serious side effects are rare when these medications are taken at moderate dosages, many of these medications can be given safely and simultaneously without the need for expensive testing and monitoring [14]–[18]. In addition, these interventions are often cost saving, even in the poorest countries [19]–[22].

China, a rapidly industrializing LMIC, faces large and growing problem of DM. In 2006, China had an estimated 92 million persons with DM, [6] 9.7% of all persons aged ≥20 years [6]. Hu et al. [23] used data from the 2003 National Health Service Investigation to estimate DM's overall annual economic burden to China at 17.6 billion Chinese yuan (CNY) in that year, about 2.7 billion U.S. dollars (USD), using a mid-2011 exchange rate. Zhang et al. [24] used a case-control study of residents of an urban neighborhood in Shanghai to estimate 2005 diabetes-caused national direct cost for medical care of CNY 39.0 billion (USD 6.0 billion). A subsequent cross-sectional study by Wang et al. [25] of patients at selected hospital clinics in four major cities proposed a much larger estimate, CNY 169.5 billion (USD 26.0 billion) in 2007 and a projected CNY 307.7 billion (USD 47.2 billion) in 2030. These and other estimates of prevalence and cost [26], [27] do not account for the majority of persons with DM in China, because their DM is undiagnosed. [6] Furthermore, these estimates were derived from sources that are either out of date- or did not cover China as a whole.

Recently, the opportunity arose to measure the impact of diabetes in China in a population-based nation-wide sample with a high response rate that also identified persons with undiagnosed diabetes. In 2007–2008, the China National Diabetes and Metabolic Disorders (ChiNDaMeD) Study tested and interviewed 46,239 persons in randomly selected sampling units in urban districts and rural townships from across China [6]. These interviewees represented 87.3% of all persons contacted to participate. Participants were tested and assigned to one of three groups: persons with DM, defined as a fasting glucose score of ≥126 mg/dL, a 2-hour glucose score of ≥200 mg/dL, or both; persons with normal glucose tolerance (NGT), defined as a fasting glucose score of ≤110 mg/dL and a 2-hour glucose score of <140/dL; and persons with impaired glucose regulation (IGR), defined as those who had neither DM nor NGT. In 2009–2010, for the present Diabetes Impact Study, we re-interviewed participants in the ChiNDaMeD study to measure the economic impact of DM on health care systems and families in China and to describe the medical care now received by Chinese people with DM.

## Methods

### Sample Selection

Seventeen of the ChiNDaMeD study sites were invited to participate and 12 agreed to do so. Each participating site was asked to sample three groups of up to 150 subjects each, from among persons previously identified to have DM, IGR, or NGT. To minimize sampling bias, each center divided potential participants from each group into sampling strata by age and sex and then sorted alphabetically by family name within each stratum. The first person in alphabetical order from each list was contacted by mobile phone and invited to come to a hospital endocrinology department to be interviewed. Participation was voluntary. Names were called until a sufficient number had agreed to be interviewed or until all potential candidates in all strata had been contacted. The participating centers delegated ethical review to the ethics committee at the Chinese-Japanese Friendship Hospital in Beijing, which approved and monitored the study.

### The Interview

The 25-page interview questionnaire ( File S1) was first developed in English and French versions, tested, and finalized for use in five African countries. For use in China, the African questionnaire was translated into Mandarin and then back-translated into English and modified some questions to better fit the Chinese medical care system. The Chinese version was then tested for validity and clarity in a sample of Chinese respondents and finalized after minor changes.

The interview included questions intended to elicit information about: the participant's use of Western and traditional medical services; payments made at the time of service for medical care services; medicines currently in the participant's possession and details about the use, cost, and acquisition of these medicines; and the recent availability and receipt of medicines and medical care for hyperglycemia, dyslipidemia, and hypertension. To obtain further details about the nature and cost of medical care encounters, interviewers asked in-depth questions about each participant's most recent overnight inpatient hospitalization (admission), if admitted during the preceding year, and most recent outpatient visit (OPV) to a hospital-based clinic or to a provider not based in a hospital, e.g., a doctor's office, if the visit occurred during the preceding three months. All interviews were conducted by physicians who had been trained in a central location and who, prior to starting the interview, verbally confirmed each subject's informed voluntary consent to participate.

### Data Entry and Verification

Interview answers were recorded on a paper form (File S1) and subsequently entered electronically using a program developed in Epi Info [28]. Because this program did not allow verification by duplicate data entry, data entry was initially verified by randomly selecting a subsample of participants from each site, then visually comparing the entered data with hard-copy records to confirm the reliability of the data entry process. After this confirmation, the entire database was searched electronically for data that were out of range or inconsistent with other variables. Suspect values were either corrected from the hard-copy records or excluded from the analysis dataset if an appropriate response could not be determined from the answers in the hard-copy interview form.

### Estimation of Expenditures for Medical Care

The study team relied on participant recall to ascertain charges for medicines, supplies, and medical care services. To increase accuracy of recall, the interview schedule asked about events occurring only during the previous 90 days and attempted to improve temporal accuracy by asking respondents to name and associate a well-remembered event that had occurred approximately 90 days previously. To estimate expenditures for medicines, interviewers asked subjects about their most recent purchase of each of the medicines they were currently using. For overnight admissions to hospital and OPVs, respondents were asked to recall their total point-of-service payment, including charges for medicines and tests received or, if they paid only a portion out of pocket, the total bill or charge. Many insured participants said that they had paid the total charge at the time they had received the medical care, although insurance agencies later reimbursed that money, in whole or in part. Some others with insurance paid only the copayment amounts but knew the total charges, because these were recorded in medical record booklets that they took to every medical encounter (and were asked to bring to the study interview). Total charges per visit or hospital admission usually included charges for medicines because Chinese doctors and hospitals typically use pharmaceutical sales as a primary means to finance operations.

Because details about only the most recent medical encounters and transactions of each type were obtained, the study team used this subset to estimate the characteristics of all events of the same kind, including mean length of hospital stay and mean payments per admission, per OPV, and per purchase of medicine. Using these averages, the study team then calculated quarterly statistics for each of four subgroups: urban persons with DM, rural persons with DM, urban persons with NGT, and rural persons with NGT. For this paper, data collected from persons with IGR (blood glucose results in the pre-diabetic range) were not analyzed.

### Calculation of Annual Medical Expenditures

Annual rates of use of medical services were calculated by multiplying the amounts self-reported for the preceding 90 days by 4.0. The study team calculated 90-day OPV expenditures separately for visits made by a participant to hospital clinics, to the private offices of doctors of Western medicine, to doctors of traditional Chinese medicine, and to community health workers. Although participants were asked about visits to hospital emergency wards, too few such visits were reported to support analysis, probably because emergency room visits were defined to exclude any visit resulting in an overnight stay. Expenditures for diabetes education could not be ascertained because educators did not practice outside of hospital clinics and the cost for the educator could not be separated from overall hospital costs.

To estimate expenditures for medicines, the study team asked detailed questions about each medicine that a person was currently using. Mean expenditures for medicines were calculated separately by where the medicine was obtained (hospital pharmacy vs. elsewhere), where the person lived (urban vs. rural), whether the person had DM or NGT, and what class of medicine was purchased (metformin, sulphonylurea, acarbose, insulin, some other antihyperglycemic, an antihypertensive, a lipid-lowering medicine, an antithrombotic, or “other,” for any prescribed medicine not falling into one of the eight previous categories). Within each of the resulting 2×2×2×9 = 72 strata, we calculated an average daily price, using data about the price paid the most recent time the item was purchased, the number of pills or units of insulin purchased at that time, and the number of pills or units prescribed per day. We then multiplied this result by an adjuster for self-reported adherence, the average number of days per week that the participant indicated that he or she adhered to the prescribed regimen for a given medicine, divided by seven. This gave us a payment per day “as used.” Mean daily payments were multiplied by 30 to obtain a monthly mean expenditure and by 365 to obtain an annual mean expenditure. File S2, Table S2–3 displays the calculated mean annual expenditures for diabetes-related and non-diabetes-related “Western” medicines by source (hospital, private pharmacy and other) and by diabetes status, plus expenditure ratios. File S2, Table S2–4 provides a detailed breakdown of annual payments for individual classes of diabetes-related medicines by urban vs. rural location in public and private pharmacies, also by diabetes status. Payments for glucose testing strips were calculated similarly to payments for medicines except that self-reported testing rates (times per day x days per week or month) were used in lieu of prescribed usage rates and adherence to obtain mean daily, monthly, and annual usage and expenditure.

We calculated total expenditures for medical care per person as the sum of estimated annual payments per person for inpatient hospital admissions, annual payments per person for outpatient services, annual payments per person for medicines, and annual payments per person for glucose-testing supplies. To avoid double-counting, because self-reported payments for OPVs and admissions included payments for medicines, the study team subtracted from the grand total payments for medicines and strips that were purchased from hospitals during visits and admissions. To calculate the amount to subtract, the team first estimated the mean “as-used” supply (in days) of medicines, including aspirin and other over-the-counter products, when purchased from hospital pharmacies (approximately 32 days, data not shown). Based on patient self-report, we then calculated the proportion of hospital clinic OPVs during which medicines were purchased (0.737 for persons with DM, 0.606 for persons with NGT). Because the interview schedule did not ask subjects which medicines they purchased at OPVs, we assumed (conservatively, as participants could also purchase medications from independent pharmacies) that one refill of every current medicine was purchased at every OPV. The team used the same procedure to remove the double-counting of medicine purchases during inpatient stays, except that here we assumed that medicines were purchased at every admission and that hospitalized patients purchased a 14-day supply, which is the policy in all public Chinese hospitals. (File S2, Tables S2–3 and S2–4 show the costs of medicines when purchased at hospital pharmacies).

### Testing the Representativeness of the Samples

To assess the extent to which each sample was representative of the larger population-based sample from which it was drawn, the study team linked subject identification (ID) numbers in its dataset to ID numbers in the ChiNDaMeD database after stratifying by location, DM status, and sex; and then matching on name, followed by address, telephone number, and approximate age. This re-matching procedure was needed because not all sites recorded the original ChiNDaMeD IDs on their interview forms. The study team was able to match 80.2% of participants using this method. To assess the statistical significance of differences between our study sample and the source sample, the matched ChiNDaMed samples of persons with DM and with NGT were compared to all the other participants in the ChiNDaMed at our 12 study sites. Chi-squared tests were used to find the statistically significant differences.

### Hypotheses and Hypothesis Testing

The study team's *a priori* primary hypothesis was that total annual per-person expenditures for medical care among persons with DM would exceed total annual per-person expenditures among persons with NGT, after adjustment for differences in age, sex, and urban vs. rural residence. This hypothesis could not be tested directly using standard statistical procedures because, as described above, calculating total expenditure required us to combine person-level data with service-level and medicine-level data; and because, for services and medicines we had data on only a subset (the most recent) of all the services and medicines that our subjects purchased. The study team therefore elected to apply formal statistical tests only to the underlying “natural” statistics in each of the various datasets (person-, use-, and medicine-level). Mean rates of use of services per person, mean length of stay per hospital admission, and mean payments per purchase of a medicine are examples of such natural statistics.

Analysis of many of these natural statistics was further complicated by the large number of persons who indicated a value of zero or provided no data on key variables, either because they had no admission or doctor visit during the preceding 90 days or because they were not taking any medicine at the time of the interview. To cope with this, the study team used a two-step “hurdle” model [29] to test for differences between persons with DM and persons with NGT. Using multivariable logistic regression models that included case status (with DM  = 1, with NGT  = 0), the team first tested for differences in the proportion of subjects with a non-zero value, e.g., persons with any OPV during the preceding 90 days, controlling for continuous age, sex, and urban vs. rural residence as well as case. Age was entered as a linear continuous variable because the addition of other transformations on age, e.g., age squared, did not significantly improve the performance of the model. Then, the team estimated a second, identically specified multivariable model on the non-zero values. The functional form of the second regression model depended on the underlying distribution of values: for counts of admissions, OPVs, and medicines, a Poisson regression model was used, while for average length of hospital stay and costs per event, we used ordinary least squares. The hurdle approach does not yield a single overall coefficient or confidence interval for hypothesis testing. However, if the coefficient on DM status is positive and significantly different from zero in one model and positive and not significantly different from zero in the other model, or if both models are significantly positive for DM, then the two models together may be considered statistically significant. Because our primary hypothesis was one-sided, we used one-sided tests (p<0.10) to assess significance. The study team considered but decided not to use random-effects models to adjust for clustering within sites, because the sampling frame was directly matched by site and because our primary objective was to measure synthetic ratios of expenditure across the Chinese national sample as a whole.

### Tabulation of Healthcare Use and Expenditures

Absolute results for health care use and costs associated with DM can be estimated by subtracting age-, sex-, and location-adjusted estimates for persons with NGT from the relevant unadjusted results for persons with DM. However, for purposes of comparing results from this study to the results of other studies conducted in other places and times, a more robust statistic is the adjusted *ratio* of costs or use among persons with DM to those among persons with NGT (DM:NGT ratio). Ratios are much less influenced than absolute differences by variations in source data, recruitment bias, differences in economic systems, conditions and patterns of medical practice, and currency fluctuations [3], [30]–[32]. To conserve space, the tables in this paper always present DM:NGT ratios and usually show absolute results only for persons with DM. Absolute results for persons with NGT can be calculated by dividing the results for persons with DM by the DM:NGT ratio. The absolute amount of use or expenditure estimated to be caused by diabetes can then be obtained by subtracting an absolute amount for persons with NGT from an absolute amount for persons with DM.

As described above, we tested hypotheses about the impact of DM using after statistical adjustment sex, urban-rural residence, and continuous age. However, to support policy and managerial analyses, it is helpful to break down estimates of use and costs by subgroups. For clarity and consistency across estimates, we have tabulated these breakdowns without statistical adjustment. But in the right-most column, most tables also include adjusted estimates for the study sample as a whole. These adjusted estimates were obtained directly by calculating each NGT result to approximate the result that would have been obtained if the NGT sample had had the same age-sex distribution as the sample with DM, based on a stratification into ten groups, two for sex (male or female) and five for age (<40, 40–49, 50–59, 60–69, or ≥70 years). However, all p-values indicated in the tables, including p-values for displayed unadjusted associations with age, location, and years since diagnosis, were taken from multivariable regressions on DM status vs. NGT (when relevant), continuous age, sex, urban vs. rural residence and, for comparisons involving only persons with DM, length of time since diagnosis.

### Analysis of Use of Effective Diabetes Care

Estimates of the percentage use of essential medicines and other percentage measures of quality and access to medical care were calculated only for persons with DM. Associations with categories of age and length of time since diagnosis of DM were tested for statistical significance using multivariable logistic regression models with continuous age, sex, urban vs. rural residence, and continuous duration of DM as predictors.

### Source Data and Programs

File S3 contains links to the study data (without personal identifiers), to the R programs that we used to transform and analyze this dataset, and to the data entry program. Other scientists are free to use these resources to confirm our results and to perform and publish additional analyses.

## Results

### Recruitment

Approximately two-thirds of the subjects whom the study team contacted completed the interview. The proportion of persons who agreed to participate was greater among persons with DM than among persons with NGT. After data cleaning and verification, 1481 out of the 1533 interviews of persons with DM and 1553 out of the 1620 interviews of persons with NGT provided usable data. Persons with DM were, on average, 6.4 years older than persons with NGT (95% CI: 57.4≤13.3 years vs. 51.0±13.3 years, p<0.01) and less likely to be female (58.3% vs. 62.9%, p<0.05). The overall proportions living in urban areas were almost identical, 75.6 vs. 76.0%, although in specific sites these proportions sometimes differed. File S2, Table S2–1 details the characteristics of study participants by study site.

Table 1 uses data gathered by the original ChiNDaMeD screening survey to compare the total ChiNDaMeD sample to the 80.2% of our interviewees with complete data whom we were able to match in both datasets, as described in the Methods section. Statistical significance for the differences shown in Table 1 were calculated separately for persons with DM and for persons with NGT, comparing our interviewees to the remainder of the ChiNDaMeD sample, not to the total ChiNDaMeD sample as displayed in the table. With few exceptions, study participants with DM did not greatly differ from persons with DM in the sample from which they were drawn. Although participants were 1.3 years older on average (p<0.01) and 4.7 percentage points more likely to live in a city (p<0.01), they were not significantly more likely than the sampling frame to be women or to have a history of stroke, myocardial infarction, hypertension, heart failure, renal disease, eye disease, or peripheral artery disease; or to have had a BMI >28 kg/m^2^ or a waist circumference >80 cm or >90 cm in women and men, respectively. The major observed difference was that persons whose DM was discovered by screening were a lower proportion of the study sample (34.9%) than of the sampling frame (44.7%, p<0.01). In addition, elevated fasting plasma glucose (FPG), systolic blood pressure (SBP), and low-density lipoprotein (LDL) cholesterol were more prevalent in the sample than in the frame.

**Table 1:** Characteristics of study sample and source sample a.

	With Diabetes			With Normal Glucose Tolerance		
	Source^b^	Study^c^	p-value^d^	Source^b^	Study^c^	p-value^d^
**Population characteristics**						
N	4454	1232	NA	26875	1201	NA
Mean age	54.4	55.7	<0.01	42.7	46.9	<0.01
Percentage female	58.5	58.9	0.83	62.3	63.1	0.56
Percentage urban	71.0	75.7	0.00	63.9	77.8	<0.01
Percentage study-diagnosed^e^	44.7	35.9	0.00	0.0	0.0	NA
**Medical history**						
Stroke	3.3%	3.7%	0.31	0.8%	1.3%	0.05
MI	1.8%	1.8%	0.93	0.3%	0.7%	0.01
Hypertension	39.1%	41.8%	0.10	13.1%	18.6%	0.00
Heart failure	0.5%	0.2%	0.26	0.2%	0.1%	0.61
Any CVD	38.4%	41.8%	0.20	12.9%	18.8%	0.00
Renal disease	0.3%	0.4%	0.34	0.1%	0.1%	0.83
**Physical measurements**						
FPG >8 mmol/L	35.7%	41.1%	0.00	0.0%	0.0%	NA
SBP ≥140 mmHg	23.3%	26.1%	0.05	10.5%	12.8%	0.01
LDL ≥3 mmol/L	46.6%	52.5%	0.00	31.2%	39.9%	0.00
BMI ≥28 kg/m2	14.7%	15.4%	0.58	8.4%	7.4%	0.21
Waist (women) ≥80 cm	72.9%	73.8%	0.56	41.4%	42.3%	0.56
Waist (men) ≥90 cm	53.2%	51.6%	0.43	30.2%	30.1%	0.96

BMI =  body mass index, CVD =  cardiovascular disease, FPG =  fasting plasma glucose, LDL =  low-density lipoprotein cholesterol, MI =  myocardial infarction, N =  number of participants, SBP =  systolic blood pressure, Waist =  waist circumference, NA =  not applicable.

a The source sample is the participants in the 2007–2008 ChiNDaMeD population-based screening study.

b “Source” refers to the ChiNDaMed source sample.

c “Study” refers to the 80.2% of participants in the present study who could be matched to a person in the ChiNDaMed dataset (the source sample).

d p-values were calculated using Chi-squared tests that compared the study sample with all subjects in the source sample who could NOT be matched or were NOT included in the study sample.

e “Study-diagnosed” means that the participant had not been diagnosed prior to, or did not recall having been diagnosed at the original ChiNDaMeD interview, but was diagnosed by a fasting plasma glucose test by the ChiNDaMeD study.

Study participants with NGT on the first survey were substantially older and sicker than their counterparts in the sampling frame. They were 4.2 years older (46.9 years vs. 42.7 years, p<0.01), 13.9 percentage points more likely to be urban (77.8% vs. 63.9%, p<0.01), and statistically significantly more likely to have had one or more strokes, myocardial infarction, hypertension, or at least one cardiovascular problem. Similarly to the participants with DM, participants with NGT were more likely to have had elevated SBP and LDL than non-participant NGTs.

### Use of Inpatient Care

As shown in Table 2, 3.60% of persons with DM reported at least one admission during the preceding 90 days, compared to 1.61% of persons with NGT, 1.90% after direct adjustment for age, sex, and location. The 90-day data extrapolated to 11.2% of persons with DM having had at least one hospital admission during the preceding year, assuming, based on data not shown that 26.0% of subjects who an admission during one quarter had another admission during the other nine months of the year. After adding in multiple admissions within each 90-day period, we calculated that each person with diabetes averaged 0.19 admissions per year, and that persons with NGT experienced raw and age-and sex-adjusted rates of 0.08 and 0.10 admission, respectively. The higher number of admissions was attributed largely to “diabetes” and heart disease (File S2, Table S2–5 for details).

**Table 2:** Use of inpatient services by location, age range, and length of time since diagnosis.

	Location		Age in Years					Years since Diabetes Diagnosis				All Participants	
	Urban	Rural	<40	40–49	50–59	60–69	≥70	<2	3–5	6–10	>10	Raw	Adjusted
N (DM)	1121	355	88	259	459	398	246	527	325	280	146	1481	1481
N (NGT)	1154	392	322	344	445	283	133					1553	1553
**Estimated percentage of participants with** ≥**1 inpatient admission, preceding 90 days**													
DM	3.57	3.66	1.14	2.70	2.40	4.52	6.50^a^	3.04	3.08	3.57	8.90^c^	3.58	3.58
NGT	1.73	1.28	0.93	1.16	0.90	3.89	2.26^b^					1.61	1.93
Difference	1.83	2.39	0.20	1.54	1.50	0.64	4.25					1.97	1.65^d^
Ratio	2.06	2.87	1.22	2.32	2.67	1.16	2.88					2.22	1.85
**Mean number of admissions, preceding 90 days, among participants admitted**													
DM	IO	IO	IO	IO	IO	IO	IO	IO	IO	IO	IO	1.30	1.30
NGT	IO	IO	IO	IO	IO	IO	IO					1.24	1.25
Difference	IO	IO	IO	IO	IO	IO	IO					0.06	0.05
Ratio	IO	IO	IO	IO	IO	IO	IO					1.05	1.04
**Mean length of stay, most recent hospital admission, among participants admitted**													
DM	13.51	12.31	8.00	18.00	11.14	13.00	12.24	10.90	10.25	18.50	14.43^c^	13.22	13.22
NGT	11.71	20.43	12.60	16.29	11.80	14.07	8.00					13.46	12.93
Difference	1.80	−8.12	−4.60	1.71	−0.66	−1.07	4.24					−0.23	0.29
Ratio	1.15	0.60	0.63	1.11	0.94	0.92	1.53					0.98	1.02
**Mean annual inpatient days per person, all participants** e													
DM	2.61	2.09	0.36	2.22	1.17	3.81	3.80	1.49	1.40	2.64	9.95	2.47	2.47
Ratio	2.47	2.01	0.46	2.94	2.21	1.36	5.26					2.30	1.98
**Mean annual inpatient admissions per person, all participants** f													
DM	0.19	0.17	0.05	0.12	0.11	0.29	0.31	0.14	0.14	0.14	0.69	0.19	0.19
Ratio	2.14	3.33	0.73	2.66	2.34	1.48	3.44	0.00	0.00	0.00	0.00	2.34	1.93

DM diabetes mellitus, IO insufficient observations to calculate the statistic, N number of participants, NGT normal glucose tolerance.

a p-value ≤0.10 for age.

b p-value ≤0.05 for age.

c p-value ≤0.01 for duration of diabetes.

d p-value ≤0.05 for overall DM vs. NGT difference.

e p-values not calculated for this category because results were derived in part from means instead of person-level data.

f p-values not calculated for this category; p-values calculated for components within the category.

**General notes**

1) Data based on use of services during the preceding 90 days, as reported by the participant, except *mean length of stay*, which was based on the most recent hospitalization during the preceding year.

2) *Difference* was defined as the rate for participants with DM minus the rate for participants with NGT.

3) *Ratio* was defined as the rate for participants with DM divided by the rate for participants with NGT.

4) *Mean number of admissions among those admitted, preceding 90 days* was not broken down by number of admissions because multiple hospitalizations occurred too infrequently to yield meaningful results.

5) *Mean annual admissions per person* were determined by multiplying self-reported admissions per person from preceding 90 days by 4.0.

6) *Mean inpatient days per person* were determined by multiplying mean annual admissions/person by mean length of stay.

7) P-values were intentionally not calculated for the *days per person* and *admissions per person* data because these values were calculated from group means.

8) Results as displayed are unadjusted except for the NGT values in the column labeled “All Participants/Adjusted.” In that column, adjusted values for participants with NGT were directly standardized to the age-sex distribution of participants with DM.

9) All p-values were calculated from multivariable regression models using continuous measures of age and, for participants with DM, years since diabetes was diagnosed.

**Table 3:** Use of outpatient services by location, age range, and length of time since diagnosis.

	Location		Age in Years					Years since Diabetes Diagnosis				All Participants	
	Urban	Rural	<40	40–49	50–59	60–69	≥70	<2	3–5	6–10	>10	Raw	Adjusted
N (DM)	1121	355	88	259	459	398	246	527	325	280	146	1481	1481
N (NGT)	1154	392	322	344	445	283	133					1553	1553
**Estimated percentage of participants with ≥1 outpatient visit, preceding 90 days**													
DM	49.64	34.84^a^	19.54	34.88	45.09	51.52	61.16^b^	42.99	44.55	54.68	69.29^c^	46.10	46.10
NGT	24.65	19.13^a^	16.82	18.31	25.62	29.33	30.30^b^					23.28	25.39
Difference	24.98	15.71	2.72	16.57	19.47	22.19	30.85					22.82	20.71^d^
Ratio	2.01	1.82	1.16	1.90	1.76	1.76	2.02					1.98	1.82
**Mean outpatient visits per participant with ≥1 outpatient visit, preceding 90 days**													
DM	4.09	2.87	2.47	3.32	3.66	3.93	4.57^b^	3.22	3.67	4.56	5.36^c^	3.86	3.86
NGT	3.00	2.51	1.87	2.13	3.19	3.72	2.93^b^					2.88	2.97
Difference	1.10	0.36	0.60	1.20	0.47	0.20	1.65					0.98	0.89^d^
Ratio	1.37	1.14	1.32	1.56	1.15	1.05	1.56					1.34	1.30
**Mean annual visits to hospital outpatient departments, per person, all participants**													
DM	7.60	3.32	1.61	4.25	6.20	7.54	10.23^b^	5.04	6.14	8.80	14.43^c^	6.56	6.56
Ratio	2.79	2.04	1.40	3.04	2.07	1.84	3.59					2.69	2.33^d^
**Mean annual visits to a western medical provider, per person, all participants**													
DM	0.16	0.16	0.14	0.17	0.19	0.13	0.15	0.22	0.11	0.20	0.00	0.16	0.16
Ratio	0.96	1.93	2.20	1.62	1.12	1.03	0.37					1.11	0.88
**Mean annual visits to a traditional Chinese medicine provider, per person, all participants**													
DM	0.12	0.15	0.09	0.11	0.03	0.05	0.49	0.06	0.02	0.46	0.08	0.13	0.13
Ratio	5.00	14.35	3.66	4.65	0.73	undef	undef					6.16	0.17
**Mean annual visits to a community health worker, per person, all participants**													
DM	0.24	0.37	0.09	0.11	0.17	0.36	0.31	0.22	0.26	0.50	0.33	0.27	0.27
Ratio	5.38	1.82	3.66	3.10	2.42	2.56	1.03					3.21	2.22^d^
**Mean total annual outpatients visits to any medical provider, per person, all participants**													
DM	8.13	4.00^a^	1.93	4.64	6.60	8.09	11.19^b^	5.54	6.54	9.97	14.86^c^	7.13	7.13
Ratio	2.75	2.09	1.53	2.98	2.02	1.85	3.16					2.65	2.40^d^

DM diabetes mellitus, N number of participants, NGT normal glucose tolerance, undef undefined.

a p≤0.001 for urban vs. rural difference.

b p≤0.001 for age.

c p≤0.001 for duration of diabetes.

d p≤0.001 for overall DM vs. NGT difference except community health workers, where p<0.05.

**General notes**

1) Data based on use of services during the preceding 90 days, as reported by the participant.

2) *Difference* was defined as the rate for participants with DM minus the rate for participants with NGT.

3) *Ratio* was defined as the rate for participants with DM divided by the rate for participants with NGT.

4) *Mean annual visits per participant* were determined by multiplying self-reported visits from preceding 90 days by 4.0.

5) Results as displayed were unadjusted except for the NGT values in the column labeled “All Participants/Adjusted.” In this column, adjusted values for participants with NGT were directly standardized to the age-sex distribution of participants with DM.

6) All p-values were calculated from multivariable regression models using continuous measures of age and, for participants with DM, years since diabetes was diagnosed, estimated based on recalled data from the preceding 90 days.

7) Values were “undefined” if no use was reported by participants with NGT.

8) When not displayed, the rate for participants with NGT can be calculated by dividing the rate for participants with DM by the DM:NGT ratio.

The first stage of the hurdle model confirmed that persons with DM were more likely to have had at least one admission than persons with NGT, after adjustment for age, sex, and location (p = 0.02). In the second stage of the hurdle model, the mean number of admissions per person, if admitted during the preceding 90 days, was higher in persons with DM but did not significantly differ between persons with DM and persons with NGT, 1.30 vs. 1.24, NS, after adjustment using a multivariable zero-truncated Poisson count model. Thus, the data support the hypothesis that people in China with DM had more overnight hospital admissions than those with NGT.

Hospital stays were about the same length for persons with DM and NGT. The unadjusted average length of stay per admission was slightly shorter among persons with DM than among persons with NGT (13.22 days vs. 13.46 days, NS), but this difference was reversed after age-sex adjustment (13.22 days vs. 12.93 days, NS). The median stay was ten days for persons with DM and 12 days for persons with NGT (data not shown).

For all persons with DM in the sample, there was an average 2.47 inpatient days per year, calculated as the product of average length of stay and estimated annual admissions per person for people with DM, divided by the total number of people in the sample with DM. For persons with NGT, the corresponding figure was 1.08 days per person per year (1.25 days/year after direct adjustment). The unadjusted and adjusted DM:NGT ratios for annual inpatient days per person were 2.30 and 1.93, respectively (p<0.01 for the adjusted ratio, based on a single-stage Poisson regression model with partially imputed data and zeros included).

As detailed in Table 2, among persons with DM, annual estimated admissions and annual inpatient days per person increased with age (p = 0.06 and p = 0.13), but increased dramatically with length of time since diagnosis of DM (both p<0.001). The study team observed more than a four-fold increase in annual admissions per person and a greater than six-fold increase in inpatient days per person per year among persons whose DM had been diagnosed for >10 years compared to persons diagnosed for <5 years. Inpatient health care use differed little between urban and rural persons with DM.

### Use of Outpatient Care

Table 3 provides the self-reported use of outpatient services by persons with DM and NGT over the preceding 90 days (percentage of persons with ≥1 OPV and, among persons with at least one visit, the average number of OPVs). Nearly all ambulatory OPVs both by persons with DM and by persons with NGT were to hospital-based clinics: 6.56 out of 7.13 OPVs per person with DM; 2.44 out of 2.68 OPVs per person with NGT. Among both persons with DM and persons with NGT, <1% reported visiting a traditional Chinese medicine provider during the preceding 90 days and <2% reported visiting a provider of Western medicine outside of a hospital. Also among both persons with DM and persons with NGT, the percentage of persons with at least one OPV and the number of OPVs per person increased dramatically and steadily with age (p<0.001 and p<0.001, respectively). Among persons with DM, both the annual rate and number of OPVs per person to a hospital outpatient clinic also increased steadily with the length of time since diagnosis of DM (p<0.001 in both cases). Persons living in rural areas, especially those with DM, were much less likely to visit outpatient clinics than were people living in urban areas (38.9% vs. 49.6% among persons with DM with at least one OPV, p<0.001, and 4.00 vs. 8.13 total annual visits per person, p<0.001). The numbers of OPVs to private practice Western doctors and doctors of traditional Chinese medicine were too small to permit the study team to specify frequency of visit by patient demographic characteristics.

With respect to testing the formal hypothesis that persons with DM will have more OPVs per 90 days than persons with NGT after adjusting for age, sex, and location, the coefficient on diabetes was significant (p<0.001) in a positive direction in both the first-stage, zero hurdle logistic model and in the second-stage Poisson-regression count model, confirming the hypothesis. Details of these models are shown in File S2, Table S2–7. In their recall of the causes of visits, subjects also reported that most of the higher number of OPVs for persons with DM was attributable to “diabetes” (File S2, Table S2–6). To see median rates of use of inpatient and outpatient services, please consult File S2, Table S2–2.).

### Use of Medicines

Table 4 shows the use of Western medicines by persons with DM and by persons with NGT. (We defined a Western medicine as any compound that was not a traditional Chinese medicine.) Almost two-thirds (66.2%) of persons with DM reported taking at least one medicine at the time they were interviewed, compared to less than one-fourth (23.5% age-sex adjusted, 18.4% unadjusted) of persons with NGT. This DM vs. NGT difference was highly statistically significant (p<0.001) in the first-stage of hurdle model, controlling for age, sex, and location. In the second-stage of the model, the mean number of medicines among persons taking any medicine was also significantly different between persons with DM (1.94 medicines) and persons with NGT (1.58 medicines, 1.64 adjusted, p<0.001), supporting our *a priori* hypothesis. Use of medicines increased with age and with the length of time since diagnosis of DM (both p<0.001). Persons with DM living in rural areas were less likely than persons with DM living in urban places to be taking medication (56.9% vs. 69.0%, p<0.01). However, the number of medicines taken per person did not vary significantly with age, duration of disease, or urban vs. rural location among persons with DM who were taking at least one medicine.

**Table 4:** Use of “Western” medicines by location, age range, and length of time since diagnosis.

	Location		Age in Years					Years since Diabetes Diagnosis				All Participants	
	Urban	Rural	<40	40–49	50–59	60–69	≥70	≤2	3–5	6–10	>10	Raw	Adjusted
N (DM)	1121	355	88	259	459	398	246	527	325	280	146	1481	1481
N (NGT)	1154	392	322	344	445	283	133					1553	1553
**Estimated percentage of persons taking ≥1 Western medication**													
DM	68.96	56.90^a^	52.27	52.51	64.27	73.37	75.20^b^	59.77	72.00	81.43	92.47^c^	66.17	66.17
NGT	19.15	16.07	4.35	11.34	19.33	35.34	30.83					18.35	23.46
Ratio	3.60	3.54	12.02	4.63	3.33	2.08	2.44					3.61	2.82^d^
**Mean number of Western medicines taken, among participants taking any**													
DM	1.98	1.81	1.41	1.72	1.76	2.03	2.39^b^	1.83	1.91	2.05	2.33^c^	1.94	1.94
NGT	1.57	1.62	1.36	1.33	1.48	1.73	1.78^b^					1.58	1.64
Ratio	1.26	1.12	1.04	1.29	1.19	1.17	1.34					1.23	1.19
**Mean number of Western medicines taken per person, all participants**													
DM	1.37	1.03^a^	0.74	0.90	1.13	1.49	1.80^b^	1.09	1.38	1.67	2.15^c^	1.29	1.29
NGT	0.30	0.26	0.06	0.15	0.29	0.61	0.55^b^					0.29	0.38
Ratio	4.53	3.96	12.52	5.98	3.97	2.43	3.28					4.43	3.35^d^

DM =  diabetes mellitus, N =  number of participants, NGT =  normal glucose tolerance, undef =  undefined.

a p≤0.05 for urban vs. rural difference.

b p≤0.05 for age.

c p≤0.05 for duration of diabetes.

d p≤0.05 for overall DM vs. NGT difference.

**General Notes**

1) Data based on self-reports of the medicines that each participant using at the time of the interview.

2) *Difference* was defined as the rate for participants with DM minus the rate for participants with NGT.

3) *Ratio* was defined as the rate for participants with DM divided by the rate for participants with NGT.

4) *Western medicines* were defined as all medicines except traditional Chinese medicines.

5) Results as displayed unadjusted except for the NGT values in the column labeled “All Participants/Adjusted.” In this column, adjusted values for participants with NGT were directly standardized to the age-sex distribution of participants with DM.

6) P-values ≤0.05 include values ≤0.01<0.001. P-values were calculated from multivariable regression models using continuous measures of age and, for participants with DM, years since diabetes diagnosis, before annualization.

7) When not displayed, the rate for a participant with NGT can be calculated by dividing the rate for participants with DM by the DM:NGT ratio.

### Payment for Services

As detailed in Table 5, persons with DM reported paying 3.97 times as much (3.38 times as much, age-sex adjusted) for medical care services during the preceding year as persons with NGT. For persons with DM, payments were about equally divided between inpatient and outpatient services. However, persons with DM paid 4.97 times more (4.20 adjusted) than people with NGT for hospital services, in part because payments per admission were about twice as high for persons with DM as for persons with NGT (2.12, 2.18 adjusted, p = 0.08). In the case of payments for OPVs, the ratio of payments for participants with DM to payments by participants with NGT was less than for inpatient care but still substantial (2.85, 2.44 adjusted). Payments per OPV did not differ significantly between the groups (DM: NGT ratio 1.07, 1.03 adjusted, NS). In both groups, payments for medicines constituted a somewhat lower proportion of total expenditures than payments for inpatient or outpatient services, but persons with DM paid much more for medicines annually than NPVs. Annual payments for medicines from all sources totaled CNY 1573 per person with DM, and the DM:NGT ratio was 9.73 (adjusted, 7.97).

**Table 5:** Payments/charges for medical care by location, age range, and length of time since diagnosis (2009 Chinese yuan).

	Location		Age in Years					Years since Diabetes Diagnosis				All Participants	
	Urban	Rural	<40	40–49	50–59	60–69	≥70	≤2	3–5	6–10	>10	Raw	Adjusted
**Mean payment per inpatient admission, if admitted**													
DM	16,204	3643^a^	2400	18,605	18,200	10,922	11,344	15,388	8294	17,313	13,849	13,513	13,513
Ratio	2.77	0.45	0.20	3.80	4.16	1.66	2.62					2.12	2.18^d^
**Total annual payment per person for inpatient care** f													
DM	3131	619	109	2299	1912	3199	3519	2102	1130	2473	9551	2527	2527
Ratio	5.93	1.49	0.14	10.11	9.71	2.45	9.00					4.97	4.20
**Mean payment per outpatient visit**													
DM	337	399^a^	101	293	367	299	445^b^	283	297	371	459^c^	353	353
Ratio	1.05	1.06	0.47	1.41	0.91	0.79	1.23					1.07	1.03
**Total annual payment per person for outpatient care** f													
DM	2590	1384	163	1262	2287	2335	4595	1444	1865	3337	6676	2352	2352
Ratio	2.86	2.21	0.66	4.18	1.88	1.47	4.20					2.85	2.44
**Total annual payment per person for Western medicines**													
DM	1738	1077^a^	633	1137	1441	1840	2200^b^	1002	1645	2455	3843^c^	1573	1573^e^
Ratio	10.89	5.51	46.03	19.12	7.31	5.45	5.93					9.37	7.97
**Total annual payment per person for glucose testing strips**													
DM	74	19^a^	36	26	63	86	62^b^	19	81	96	188^c^	60	60
**Reduction for payments for medicines and testing strips duplicated in payments for hospital OPVs and inpatient admissions** f													
DM	−787	−280	−50	−257	−451	−1013	−1492	−302	−514	−1288	−4004	−640	−640
NGT	−27	−9	−1	−4	−24	−88	−48					−21	−27
**Total annual point-of-service payments/charges** f													
DM	6746	2820	890	4467	5251	6447	8883	4265	4207	7074	15894	5873	5873
NGT	1568	1229	1016	585	1586	3144	1807					1480	1737
Difference	5178	1590	−126	3883	3665	3303	7076					4393	4135
Ratio	4.30	2.29	0.88	7.64	3.31	2.05	4.91					3.97	3.38

DM diabetes mellitus, N number of participants, NGT normal glucose tolerance, OPV outpatient visit.

a p≤0.05 for urban-rural difference.

b p≤0.05 for age.

c p≤0.05 for duration of diabetes.

d p≤0.10 for DM vs. NGT difference.

e p≤0.001 for DM vs. NGT difference.

f p-values not calculated for this category because results are derived in part or entirely from means instead of person-level data.

**General notes**

1) Data based on total payments made as recorded or recalled by the participant, for the most recent instance of each kind of service or purchase. These payments do not reflect any subsequent reimbursement or subsidy.

2) *Difference* was defined as the rate for participants with DM minus the rate for participants with NGT.

3) *Ratio* was defined as the rate for participants with DM divided by the rate for participants with NGT.

4) *Western medicines* were defined as all medicines except traditional Chinese medicines.

5) Total annual payments for inpatient and outpatient care include payments for medicines and tests that the patient made at the time of the admission or visit. Total annual payments per person for Western medicines and glucose testing strips also include these payments. The reduction for duplicated payments removes this double-counting in the calculation of total annual point of service payments/charges (see text for details).

6) Adjusted values for “All Participants” who were NGT were directly standardized to the age-sex distribution of those with DM, using the age categories displayed in the table.

7) p-values listed as ≤0.05 include values ≤0.01 and lower.

8) When mean payments for participants with NGT are not shown they can be calculated by dividing the payment for participants with DM by the DM:NGT ratio.

Urban persons with DM paid nearly five times as much for hospital care as rural persons with DM, mostly because their payments per admission were higher. They paid about twice as much per year for outpatient care, because urban persons with DM made about twice as many visits.

Annual costs for inpatient care, outpatient care, and total care were all dramatically higher after ≥10 years of diagnosed DM: 5.52 times as much for hospital care, 4.16 times as much for outpatient care, and 3.75 times as much in total for medical care as those diagnosed ≤5 years. Expenditures in these categories also increased with age for persons with DM and NGT but, after age 40, less dramatically than for year since diagnosis. Because of four admissions involving young adults with NGT living in rural areas, who experienced long stays and high costs per admission, the ratios for inpatient costs among persons with DM compared with those for people with NGT were relatively low among rural residents and among subjects aged <40 years. This is likely to be a statistical anomaly resulting from a combination of small sample sizes in these subgroups (especially for young adults with DM), the low frequency of admissions in general (again, especially in young adults), and the generally non-normal distribution with high outlier values of inpatient length of stay and costs.

### Use of Essential Medicines and Tests

Among persons with DM, 54.8% reported not taking any glucose-lowering medicine at the time of interview (Table 6). Over two-third of persons (76.1%) with DM aged <40 years did y not use a glucose-lowering medicine, as were persons whose DM had been discovered only in the preceding one to two years (68.0%). Antihyperglycemic medicine use was higher among persons with DM in urban (48.1%) than in rural (36.3%) areas. The most commonly used glucose-lowering medicine was metformin (22.3%), followed by a sulphonylurea (16.0%), insulin (9.2%), and acarbose (8.4%). Very few persons with DM (1.8%) reported using any statin; 21.1% indicated taking an antihypertensive agent, usually a calcium channel blocker (11.6% of all persons with DM); 22.4% reported taking a daily aspirin, with only 0.5% reporting any other anticoagulant.

**Table 6:** Estimated percentage of participants with diabetes currently using essential basic diabetes medicines by location, age range, and length of time since diagnosis.

	Location		Age in Years					Years since Diabetes Diagnosis				All
	Urban	Rural	<40	40–49	50–59	60–69	≥70	≤2	3–5	6–10	>10	
N	1121	355	88	259	459	398	246	527	325	280	146	1481
Glucose-lowering agents	48.1	36.3^a^	23.9	34.0	47.5	50.0	53.3 b	32.0	54.8	65.4	80.1 c	45.2
Metformin	22.4	22.0	11.4	17.8	25.1	24.9	19.9	18.0	30.5	28.2	27.4	22.3
Sulphonylurea	17.5	11.3	9.1	12.0	15.3	17.3	22.0 b	10.6	21.2	21.1	30.8 c	16.0
Insulin	10.0	6.8	0.0	8.1	10.7	8.3	12.6	2.1	7.1	20.4	29.5 c	9.2
Acarbose	10.1	3.4 a	4.5	5.4	5.9	11.3	14.2 b	5.5	7.7	12.1	23.3 c	8.4
Other	4.3	1.7	1.1	0.4	3.9	5.0	5.7 b	2.3	4.3	6.1	7.5	3.6
Lipid-lowering agents	1.9	2.3	1.1	1.9	0.2	2.8	4.9 b	2.3	2.8	1.8	2.7	2.0
Statin	1.7	2.0	1.1	1.2	0.2	2.5	4.5 b	1.9	2.5	1.8	2.1	1.8
Other	0.4	0.3	0.0	0.8	0.0	0.5	0.8	0.6	0.3	0.4	0.7	0.4
Blood pressure–lowering agents	21.4	20.0	6.8	12.0	16.3	27.4	33.3 b	24.1	16.9	22.1	26.0	21.1
Diuretic	0.7	0.0	0.0	0.4	0.4	0.8	0.8 b	1.1	0.3	0.0	0.7	0.5
ACE Inhibitor	4.1	5.1	1.1	1.5	5.0	5.5	4.9 b	4.4	3.7	5.4	4.8	4.4
ARB	2.5	0.8	1.1	1.2	1.3	2.3	4.1 b	3.0	1.5	2.5	2.1	2.1
Beta blocker	4.4	0.8 a	0.0	1.2	3.1	4.3	6.1 b	3.8	3.1	2.9	6.8	3.5
Calcium channel blocker	12.4	9.0	3.4	6.2	9.6	12.6	22.8 b	11.7	10.2	14.3	15.1	11.6
Other	4.7	8.2 a	1.1	4.6	2.8	7.9	8.1 b	8.1	3.1	5.0	2.1 c	5.5
Anticoagulants	24.1	18.3	22.7	15.8	17.9	26.1	31.7 b	22.0	22.8	27.5	36.3 c	22.7
Aspirin	23.7	18.3	22.7	15.8	17.9	25.6	30.9 b	21.6	22.8	26.8	36.3 c	22.4
Other	0.5	0.3	0.0	0.4	0.2	0.5	1.2	0.6	0.3	0.7	0.7	0.5
Analgesics	0.4	0.6	0.0	0.4	0.0	0.5	0.8	0.9	0.0	0.4	0.7	0.5
Other Western medicines	9.1	10.1	6.8	6.2	6.5	10.8	15.9 b	10.0	8.9	8.9	14.4	9.4
Traditional Chinese medicines	16.6	18.9	13.6	11.6	15.9	19.8	19.9	17.2	13.5	23.2	25.3	17.1

ACE angiotensin converting enzyme, Angiotensin II receptor blockers, N number of participants.

a p≤0.05 for urban vs. rural difference.

b p≤0.05 for age.

c p≤0.05 for duration of diabetes.

**General notes**

1) Based on medicines in the subject possessed and was using at the time of the interview.

2) p-values listed as ≤0.05 include values ≤0.01 and lower. All p-values were calculated from multivariable logistic regressions using continuous measures of age and, for participants with DM, years since diagnosis of diabetes.

As shown in Table 6, non–diabetes-related Western medicines were being used by 9.4% of persons with DM at the time of interview. Among persons with DM, 17.1% reported using one or more traditional Chinese medicines; about half the proportion of persons with NGT (8.9%) who reported using traditional Chinese medicines (data not shown in table).

Although on average persons with DM diagnosed <5 years visited a hospital outpatient clinic about once every two months, 57.3% of persons with DM reported no contact of any kind–inpatient or outpatient–with the hospital system or private doctors within the preceding 3 months and, in rural areas, 68.5% reported no 3-month contact with a hospital. The proportion reporting hospital contact was also very low among persons with DM aged ≥20 and <40 years (17.0%). Persons diagnosed ≥10 years previously reported dramatically higher payments for medical care, as previously described, and this included an average of 14.4 OPVs per year. However, even among persons in this group, 34.9% reported no contact with hospital-based care during the preceding 90 days.

As shown in Table 7, 54.5% of persons with DM reported having had a glucose measurement at a medical office during the preceding 3 months; 48.9% recalled having had their blood pressure measured during the preceding 3 months; 32.1% recalled having had a retinal screening during the preceding year; and 17.9% recalled having had a foot examination during the preceding year. Among persons with DM, 43.3% of men and 8.7% of women said that they were current smokers.

**Table 7:** Percentage of participants with diabetes receiving other medicines and recommended services, by location, age range, and length of time since diagnosis.

	Location		Age in Years					Years since Diabetes Diagnosis				All
	Urban	Rural	<40	40–49	50–59	60–69	≥70	≤2	3–5	6–10	>10	
N	1121	355	88	259	459	398	246	527	325	280	146	1481
Any doctor visit preceding 3 months	46.2	31.5^a^	17.0	30.9	41.6	48.7	58.1^b^	39.5	40.0	51.1	65.1^c^	42.7
Any clinical visit preceding 3 months	49.6	34.8^a^	19.5	34.9	45.1	51.5	61.2^b^	43.0	44.5	54.7	69.3^c^	46.1
Blood sugar test preceding 3 months	57.1	46.5^a^	46.6	48.6	55.3	59.3	58.1	50.2	63.7	61.1	65.8^c^	54.5
Blood pressure test preceding 3 months	50.6	43.7^a^	13.6	40.5	45.5	57.3	66.3	46.8	44.3	55.4	71.9^c^	48.9
Retinal screening preceding year	36.6	18.6^a^	35.0	23.8	27.9	39.6	38.6	21.8	35.4	41.8	49.3^c^	32.1
Foot check preceding year	21.1	8.2^a^	30.8	16.2	15.2	20.5	18.0	11.0	21.4	21.5	20.1^c^	17.9
Glucose self-monitoring frequency												
<1/week	31.6	23.4^a^	26.1	28.2	32.7	30.4	27.6	24.8	36.6	37.1	33.4^c^	29.6
<1/day	20.6	9.9^a^	25.0	17.8	15.5	18.3	20.7	14.2	19.7	23.9	27.4^c^	17.9
≥1/day	16.5	2.9^a^	2.3	12.4	12.4	15.1	17.5^b^	15.0	10.8	13.9	17.1	13.1
Current smoker (All)	21.5	28.5	27.3	34.2	24.9	18.5	15.0^b^	24.6	24.7	19.4	17.1	23.2
Men	39.7	53.9^a^	42.0	55.7	52.7	35.8	23.4^b^	47.4	45.5	35.0	30.0	43.3
Women	8.8	8.6	7.9	14.3	5.9	8.1	9.9	9.0	7.9	8.0	10.4	8.7

N number of participants.

a p≤0.05 for urban vs. rural difference.

b p≤0.05 for age.

c p≤0.05 for duration of diabetes.

**General notes**

1) *Any Doctor Visit* includes visits to hospital outpatient clinics and to private doctors of Western medicine.

2) *Any Clinical Visit* includes visits to doctors, to providers of traditional Chinese medicine, and to community health workers.

3) p-values listed as ≤0.05 includes values ≤0.01 and lower. All p-values were calculated from multivariable logistic regressions using continuous measures of age and, for participants with DM, years since diagnosis of diabetes.

About half (52.6%) of persons with DM reported doing any glucose self-monitoring (data not shown); of these, 13.1% reported measuring their glucose on a daily basis and an additional 17.9% monitored less often than daily but at least once per week. Daily glucose self-monitoring was rare in rural areas (2.9%) and rare among young adults (2.3% of persons with DM aged ≥20 and <40 years). Daily monitoring did not noticeably increase with the length of time since diagnosis of DM.

## Discussion

We re-interviewed population-based samples of persons in China with DM and NGT who had been identified approximately two years earlier by the ChiNDaMeD study. From this second interview, we estimated that expenditures for medical care, based on point-of-service payments or charges, were 3.97 times higher among persons with DM than among persons with NGT, 3.38 times higher after age-sex adjustment. This ratio is dramatically higher than published ratios for similar comparisons in developed countries (range 2.0–2.4) [33]–[35]. The ratio reported from China was particularly higher than the ratio (1.7) reported for one medical care system that pursues aggressive secondary prevention of DM complications through diet, exercise, and the use of proven, low-cost generic metformin, sulphonylureas, statins, antihypertensives, insulin, and aspirin [36]. The Chinese expenditure ratio reported here is, as far as the study team is aware, the first population-based ratio from a LMIC to be published. Its size suggests that the human and economic impact of DM might be much greater in LMICs, where 75% of people with DM worldwide live, [3] than in the industrialized world where DM is diagnosed much earlier and essential effective medicines are more widely prescribed.

The result reported here is also are much higher than the estimate of medical care costs attributable to DM in China in the most recent International Diabetes Federation (IDF) *Diabetes Atlas* [3]. The latest Atlas estimate, for 2011, is USD 194 per person, based on an assumed age-sex adjusted DM:non-DM ratio of 2.0. If the adjusted expenditure ratio of 3.38 that we observed had instead been used, the IDF estimate would have been USD 656. This is very close to USD 640, the value in USD of the absolute difference in per person annual DM minus NGT costs that we observed, based on a July 1, 2011 currency exchange rate (CNY  = 0.1547 USD).

The expenditure ratios reported here imply, as do analyses using different methods, [37], [38] that in LMICs the economic burden of DM may constrain the availability of medical resources for other health conditions and retard national economic growth in future years. In China, these potential health and economic impact will be particularly large because the size of the population of persons with DM in China is growing extremely rapidly and because, as a consequence of its one-child policy, China will have a relatively small proportion of working-age people to support an aging and increasingly diabetic population [38], [39]. China's economic growth and the ongoing expansion of access to health insurance also could accelerate DM costs, by increasing the use of expensive treatments; others have projected that medical care costs for DM will increase by 50% in China over the next five years [38].

Our results also indicate that the quality and efficiency of DM care in China is not yet optimal. China has an opportunity to reduce future hospitalizations, disability, mortality, and medical care costs by diagnosing diabetes earlier and by using inexpensive generic statins, antihyperglycemics, and blood pressure medicines much more widely [40]. Evidence-based treatment guidelines from the IDF Clinical Guidelines Task Force [40] call for the use of a statin and an ACE-inhibitor or Angiotensin II receptor blocker (ARB) in all persons with type 2 DM, regardless of lipid and blood pressure levels, because of the potent cardio-protective and reno-protective effects of these medicines, alone and in combination.

The World Bank estimated that cardiovascular risk assessment, along with appropriate use of most of the medicines described above, would save 500 million disability-adjusted life years annually in China at an annual per-person cost of USD 220 per high risk individual [38]. If China fails to act quickly, the treatment costs for the 90% of Chinese persons with recently acquired DM will soon begin to approach the four-fold greater costs of the 10% of subjects in our study who had diagnosed DM for >10 years.

The situation in rural China deserves special mention. The population of rural China is aging even more quickly than that of urban China because rural-born young adults are migrating to cities and industrialized provinces to find jobs [38]. Overnight hospital usage statistics are similar between rural and urban persons with DM but outpatient attendance and medication use is much lower in rural areas. However, when preventive medicines are prescribed to them, rural residents use as many as do urban residents. Our data therefore suggest that persons with DM in rural China are less likely to receive medicines that can prevent disability and loss of life because they have less access than urban Chinese to outpatient care, and that their high use of inpatient treatment might be the unfortunate result.

This study has several limitations. It is observational, deriving estimates of the expenditures and other effects caused by diabetes from a case-control rather than an experimental comparison. Only 12 of the 17 sites from the ChiNDaMeD study conducted the follow-up interview. This, together with the ChiNDaMeD study omitting some regions, left gaps in our ability to describe the whole of China. Fortunately, in the 12 regions we included, we found few differences between those persons with DM from the ChiNDaMed study sample who did participate in this study and those who did not. The differences that emerged will have resulted in our underestimating the impact of diabetes: persons with NGT who had a history of serious health problems were more likely to agree to be interviewed and persons who were newly diagnosed, and therefore less expensive to treat, were underrepresented in the DM sample. At the same time, persons with DMs with complex medical histories were much less likely than persons with NGT with complex histories to be over-represented in the study sample. Our reliance on recall of point-of-service payments probably introduced a further negative bias. For example, many persons with DM receive glucose-lowering medicines at little or no cost as a matter of policy in China, and because Chinese hospital services are subsidized by government, so that charges do not reflect the full cost of producing medicines and services [41]. We also did not calculate or include the indirect costs of diabetes, or any non-medical direct costs, such as travel.

A unique strength of this study was its identification and inclusion of data from persons with DM who would have remained undiagnosed had not the ChiNDaMeD study found them via population-based screening (61% of the ChiNDaMeD sample, [6] 36% of our sample). In countries like China, with rapidly growing DM prevalence and developing medical care systems, the diagnosis of DM frequently occurs only after the appearance of a costly preventable diabetic complication. Our results provide an empirical basis for estimating the health care costs and services for persons with unrecognized and early diabetes, groups which had heretofore remained invisible and underrepresented in economic studies of diabetes in LMICs.

## Supporting Information

File S1 Interview Schedule.
(DOC)

File S2 Supplementary Tables. Table S2–1. Characteristics of Subjects by Study Site. Table S2.2. Median Use of Inpatient and Outpatient Services. Table S2–3. Mean annual payments (CNY) for “Western” medicines, by where purchased. Table S2–4. Detail of mean annual payments (CNY) for medicines by source, location, and DM/NGT status. Table S2–5. Reason for admission to hospital among subjects who reported an admission during the previous year. Table S2–6. Reason for visits to hospital outpatient clinics among subjects who reported a visit during the previous 90 days. Table S2–7. Results of multivariable hurdle models to test for DM vs. NGT differences in the use of overnight hospital admissions, outpatient visits, and medicines.
(DOC)

File S3 Links to Source Data, Data Entry Program, and Data Analysis Program.
(DOC)
