# Supplementary material for: Medical Care and Payment for Diabetes in China: Enormous Threat and Great Opportunity
Source: PLoS One. 2012 Sep 26;7(9):e39513. doi: 10.1371/journal.pone.0039513 (PMC3458850; doi:10.1371/journal.pone.0039513)
Supplement: File S1 — Interview Schedule. (DOC) [file pone.0039513.s001.doc]

| Supporting Information 1: Interview Schedule. This is the survey instrument that was used to conduct and record the results of the personal interviews that form the basis for: Yang W et al. (2012) Medical care and payment for diabetes in china: enormous threat and great opportunity. PLoS ONE.  **疾病影响调查** |
| --- |

| 研究代码 (通常用国家名) | | SC | 调查者代码 | IC |
| --- | --- | --- | --- | --- |
| 第1等级代码 | | L1 | 校对者: | CK |
| 第2等级代码 | | L2 | 数据录入员代码: | DE1 |
| 第3等级代码 | | L3 | 第二数据录入员: | DE2 |
| 第4等级代码 | | L4 | 病例=1, 对照 =2;  IGR=3 | CASE |
| 语言代码 | | LANG | 是否由其他人代回答?  是=1, 不=0 | SUR |
| **应答者电话:** | TEL | | **应答者ID (注意):** | ID |
| (姓) **应答者姓:** | FN | | | |
| **应答者名:** | PN | | | |

**|__|__|- |__|__|-** **|__|__|** DOI

年 月 日

**DOI: 调查日期:**  **STIM: 开始时间:**

24小时制时间

**LOC** **居住地区**/ **1** 直辖市/省会/ **2** 地级城市以上/ **3** 县/县级市/ **4 城**镇 / **5** 乡村/

LOC

SEX

**SEX 性别:** / **1**男性 / **2** 女性 /

MAR

**MAR:** **婚姻状况** / **1** 单身 / **2** 已婚 / **3** 丧偶 / **4** 分居或离婚 / **5** 同居 / **其他** /

**|__|__|__|** AGE

**AGE 年龄**:（岁）

**|__|__|__|__|-|__|__|- |__|__|-**

**年 月 日**

DOB

**DOB 出生年月:**

如果日期或者月份不清楚输入01

**DM-1: 是否有医生或者其他医务人员告诉你你有高血糖?**

DM-1

**1** 是 / **2** 不 / **88** 不知道) / **99** 拒绝回答

TERM

**TERM:如果应答者在对照组并且回答是，确认是否属实。如属实，那么终止调查.**

**1** 终止 / **0** 继续

**WRK: 下列哪种说法能最恰当的描述你的工作情况**

**1** 是 / **2** 不 / **88** 不知道/ **99** 拒绝回答

| **工作类型** | **回答** |
| --- | --- |
| 在家做家务劳动 (儿童保育, 烹饪, 打扫卫生, 照看家畜等等.) | WRK1 |
| 务农 | WRK2 |
| 个体户 | WRK3 |
| 城市农民工 | WRK9 |
| 在政府部门，事业单位工作 | WRK5 |
| 在国营企业工作 | WRK10 |
| 在私营企业(包括公益性组织)中工作 | WRK11 |
| 学生 | WRK12 |
| 我没有工作因为我的身体状况不允许工作 | WRK6 |
| 我没有工作并且正在找工作 | WRK7 |
| 我退休了或者在领养老金 | WRK8 |
| 我退休了但还在工作(例如反聘) | WRK13 |

**INET: 你家里几口人(按通常和你一起吃饭的人计算) |__|__|** INET

**INCM:你所有家庭成员的月收入估算是多少(包括各种来源)?**

家庭成员是指与应答者一起吃饭的人.

收入包括各种来源的现金和非现金收入

**|__|__|__|__|__|__|__|__|** INCM

**88** 不知道 / **99** 拒绝回答

**WTH: 你家里有下面的物品吗?**

大家庭(包括其他在一起吃饭的家里人) **1** 是/ **2** 不 / **88** 不知道 / **99** 拒绝回答

| **拥有的物品项目** | **回答** |
| --- | --- |
| 汽车 | WTH1 |
| 摩托车/拖拉机 | WTH2 |
| 自行车 | WTH3 |
| 电冰箱 | WTH4 |
| 电视机 | WTH5 |
| 计算机 | WTH6 |
| 固定电话 | WTH7 |
| 手机 | WTH8 |
| 微波炉 | WTH9 |
| 电饭锅 | WTH10 |
| 自来水 | WTH12 |
| 抽水马桶 | WTH13 |
| 私人房产(包括半产权房)added | WTH16 |
| 住房面积(added) | WTH17 |
| 耕牛added | WTH18 |

SCH

**SCH: 你的最高学历是什么?**

**1**没有上过学/ **2** 小学肄业 / **3** 小学毕业 / **4** 中学肄业 / **5** 中学毕业 / **6** 职业学校或者学徒 (例如, 美发师, 技师) ，包括中专

/ **7** 大学肄业或者大专 / **8** 大学毕业或硕士,,博士

| **健康效用指数** |
| --- |

读**:** 现在我想询问你各方面的健康状况. 当回答这些问题时，请你回想一下在在过去的一个星期中的健康状况,以及进行日常活动的能力。你可能会觉得下面一些问题中所涉及的事情与你无关，但请原谅我们要对每一个人问相同的问题。

过去的7天以前到今天，请你回顾一下你在这段时间所经历的主要活动。在选择答案时，请集中考虑在过去的一个星期中，你整体的能力，丧失的能力，和你的感觉。另外，有几个问题可能会很相似，但是它们是不同的问题，请你对每一个问题单独个别地回答。你提供的所有信息将被保密。**请如实回答.** 答案没有正确或错误之分。我们想知道的是你对自己的能力和感觉的看法。

**看** / 读整个句子, 特别是斜体字. **不要**读回答选项.

**­­­­**

HU1

**HU1**: **在过去的一周, 在没有戴眼镜或隐形眼镜的情况下你能看清楚普通报纸吗?**

**1** 是 **(*转到 HU-4*)** / **2** 不 / **88** 不知道 / **99**拒绝回答

HU2

**HU2: 在戴眼镜或者隐形眼镜的情况下，你能看清楚普通报纸吗?**

**1** 是 **(*转到 HU-4*)** / **2** 不 / **88** 不知道或者不戴眼镜，隐形眼镜 / **99** 拒绝回答

**HU3: 在过去的一周，你能看见东西吗?**

HU3

**1** 是 / **2** 不 **(*转到 HU-6*)** / **88** 不知道或者不戴眼镜，隐形眼镜 / **99** 拒绝回答

HU3A delete

**HU4: 在过去的一周，在不戴眼镜或者隐形眼镜的情况下，你能看清楚且能认出马路对面的朋友吗?**  **1** 是 **(*转到 HU-6*)** / **2** 不 / **88** 不知道 / **99** 拒绝回答

HU4

HU5

**HU5**:**在戴眼镜或者隐形眼镜的情况下，你能看清楚且能认出马路对面的朋友吗?** **1** 是 / **2** 不 **(*转到 HU-6*)** / **88** 不知道或者不戴眼镜，隐形眼镜 / **99** 拒绝回答

**听**

**HU6**: **在过去的一周, 在*没有*助听器的情况下，你和三个或三个以上的人一起谈话时，你能听清楚谈话内容吗？**

HU6

**1** 是 (***转到HU-11***) / **2**不/ **88** 不知道 / **99** 拒绝回答

**HU7**: **在*有*助听器的情况下，你和三个或三个以上的人一起谈话时，你能听清楚谈话内容吗？** **1** 是 *(****转到 HU-9***) / **2** 不 / **88** 不知道/没有戴助听器 / **99** 拒绝回答

HU7

HU8

**HU8**: **在过去的一周，你能听见声音吗?**

**1** 是/ **2** 不 (转到***HU-11***) / **88** 不知道/**99** 拒绝回答

HU9

**HU9**: **在过去的一周，在*没有*助听器的情况下，和另外一个人在安静的房间谈话时,你能听清楚谈话内容吗? 1** 是 ***转到 HU-11***) / **2** 不/ **88** 不知道/ **99** 拒绝回答

HU10

**HU10**:**在*有*助听器的情况下，和另外一个人在安静的房间谈话时,你能听清楚谈话内容吗?** **1** 是 /**2** 不 / **88** 不知道/不戴助听器/**99** 拒绝回答

**说_____________________________________________________________________________________**

HU11

**HU11:** **在过去的一周，使用你常用的语言或方言和当地陌生人谈话时，对方能*完全*听懂你所说的话吗?** **1** 是 (***转到 H-16***) /**2** 不 / **88** 不知道 /**99** 拒绝回答

**HU12: (**与当地陌生人交谈时，)**对方能听懂*部分*你说的话吗？**

HU12

**1** 是 /**2** 不/ **88** 不知道 / **99** 拒绝回答

HU13

**HU13:** **在过去的一周，在你和熟悉你的人谈话时，对方能*完全听懂*你所说的话吗?** 1 是 (***转到*** ***HU-16***)/2 不/**88** 不知道/ **99** 拒绝回答

HU14

**HU14:** (当与很熟悉你的人谈话时)**，对方能听懂*部分*你说的话吗?**

1 是 (***转到*** ***HU-16***) / 2 不 / **88** 不知道 / **99** 拒绝回答

HU15

**HU15:** **在过去的一周，你能够说话吗?**

**1** 是 / **2** 不 / **88** 不知道 / **99** 拒绝回答

**活动**

**HU16:** **在过去的一周, 在没有他人或者走路辅助器械(指机械支持物，例如支架，拐杖或助行器)的帮助下，**

HU16

**你能没有任何困难地完成弯腰，举物，跳跃，跑步的活动吗?**

**1** 是 (***转到*** ***HU-24***)/ **2** 不 / **88** 不知道 / **99** 拒绝回答

HU17

**HU17: 在没有他人或者走路辅助器械的帮助下，你能在住所附近毫无困难地走动吗?** **1** 是 (***转到*** ***HU-24***) / 2 不 / **88** 不知道/ **99**拒绝回答

**HU18:** **在没有他人或者走路辅助器械的帮助下，你在住所附近走动有困难，可还是能走动?**  **1** 是 (***转到*** ***HU-24***)/ **2** 不/ **88** 不知道/ **99** 拒绝回答

HU18

HU19

**HU19:** **在过去的一周，你能走动吗？**

**1** 是/ **2** 不 (***转到*** ***HU-22***)/ **88** 不知道 **99** 拒绝回答

HU20

**HU20:** **你在住所附近走动的时候是否需要走路辅助器械的帮助, 例如支架、拐杖或助行器?** **1** 是/ **2** 不/ **88** 不知道/**99** 拒绝回答

HU21

**HU21: 你走动的时候是否需要他人的帮助?**

**1** 是/ **2** 不/ **88** 不知道/ **99** 拒绝回答

HU22

**HU22:** **你是否需要轮椅才能在住所附近走动?**

**1** 是/ **2** 不/ **88** 不知道/没有轮椅/ **99** 拒绝回答

**HU23:** **你坐轮椅活动的时候是否需要他人的帮助?**

HU23

**1** 是/ **2** 不/ **88** 不知道/没有轮椅/ **99** 拒绝回答

**手与手指**

**HU24: 在过去的一周，你是否能够完全自如运用你的双手和十个手指?**

HU24

**1** 是 (***转到*** ***HU-28***)/ **2** 不/ **88** 不知道/ **99** 拒绝回答

HU25

**HU25:** **是否由于你的手或者手指的功能不健全，你需要他人的帮助?**

**1** 是/ **2** 不 (***转到*** ***HU-27***)/ **88** 不知道/ **99** 拒绝回答

**HU26:** **你的一些日常活动、大多数活动还是所有活动需要他人的帮助?**

HU26

**1** 一些/ **2** 大部分/ **3** 全部/ **88** 不知道 **99** 拒绝回答

**HU27:是否由于你的手或者手指的功能不健全，你在穿衣或者吃饭的时候，需要借助一些特殊工具**

HU27

**(指系衣服纽扣的勾子，打开罐子或举起小物品的抓握工具，以及其他用来克服手或手指残障的工具)**

**1** 是/ **2** 不/ **88** 不知道 / **99**. 拒绝回答

**自理**

HU28

**HU28: 在过去的一周，你能够正常地进行吃东西，洗澡，穿衣服，和上厕所等基本活动吗?** **1** 是 (***转到*** ***HU-31***)/ **2** 不/ **88** 不知道/ **99** 拒绝回答

HU29

**HU29: 你在吃东西，洗澡，穿衣服，和上厕所的时候是否需要他人的帮助?**

**1** 是 **2** 不/ **88** 不知道/ **99** 拒绝回答

**HU30: 你在吃东西，洗澡，穿衣服，和上厕所的时候是否需要特殊器械帮助?**

HU30

**1** 是 **2** 不/ **88** 不知道/ **99** 拒绝回答

**情绪**

HU31

**HU31: 在过去的一周，你感觉快乐还是不快乐?**

**1** 快乐/ **2** 不快乐 (***转到*** ***HU-33***) / **88** 不知道/ **99** 拒绝回答

HU32

**HU32: 你快乐的程度如何?**

**1** 快乐觉得生活有乐趣, (***转到*** ***HU-34***)/ **2** 有点快乐, (***转到****HU34) /***88** 不知道/ **99** 拒绝回答

**HU33:** **你不快乐的程度如何？**

HU33

**1** 有点不快乐/ **2** 非常不快乐,觉得活着没什么意思/ **3** 不知道/ **4** 拒绝回答

**HU34:** **在过去的一周,你是否有烦躁, 生气，容易发脾气, 焦虑或者抑郁的情绪?**

HU34

**1** 是/ **2** 不 (***转到*** ***HU-37****/* **88** 不知道 (***转到****HU37)/* **99**拒绝回答

HU35

**HU35:** **你出现烦躁, 生气，容易发脾气, 焦虑或者抑郁的情绪的频率如何?**

**1** 很少/ **2** 偶尔/ **3** 经常/  **4**几乎总是/ **88** 不知道/ **99** 拒绝回答

HU36

**HU36:** **在过去的一周,你是否有极端烦躁, 生气，愤怒, 易怒, 焦虑或者抑郁的情绪，以至于需要看医生?**

**1** 是/ **2** 不/ **88** 不知道/ **99** 拒绝回答

**记忆力 _______________________________ _____________________________________**

HU37

**HU37: 在过去的一周，你怎样描述你的记忆力?**

(可用探究，**你能记住多少事情？是决大多数记得住，有点健忘呢，还是一点也记不住？)**

**1** 能记住大多数的事情/ **2** 有点健忘/ **3** 非常健忘 **4**无法记住任何事情/ **88**不知道/ **99** 拒绝回答

**思考____________________________________________________________________**

HU38

**HU38:** **在过去的一周，你怎样描述你思考和解决日常问题的能力?**

**1** 能清楚的思考和解决日常生活中的问题/ **2** 有一点点困难/ **3** 有一些困难/ **4** 有很大困难/

**5** 无法思考并解决日常生活中的问题/ **88** 不知道 / **99** 拒绝回答

**疼痛或不适_________________________________________________________**

HU39

HU399

**HU39:** **在过去的一周，你有过疼痛或者不舒服吗?**

**1** 有/ **2** 没有 *(****转到*** *HU41)* / **88** 不知道 *(看 HU41)/* **99** 拒绝回答

HU40

**HU40:** **在过去的一周，由于疼痛或不舒服，你有多少活动受到影响?**

**1** 没有 **2** 少数/ **3** 一些/ **4** 大多数/ **5** 所有/ **88** 不知道/ **99** 拒绝回答

**HU41: 总体上说，你如何评价你在过去一周中的健康状况? (**可用探究**，好还是差，程度如何？)**

HU41

**1**非常好/ **2** 很好/ **3** 好/ **4** 一般/ **5** 差/ **88** 不知道/ **99** 拒绝回答

| **医疗服务的使用状况** |
| --- |

读**: 现在我要询问你过去三个月中医疗卫生服务的使用情况. 现在请开始回忆一件三个月前发生的事情. 我的大部分问题都是关于从那个时间开始你医疗卫生服务的使用情况. 你能想起三个月（90天）前你做的或者发生在你身上的一件事情或者特殊日子吗?**

HV90

**HV90: 在过去的90天中, 你去过任何一家*医院*去为你自己看病吗?**

**1** 是/ **2**.不 (***跳到*** **NONHOS**)/ **88** 不知道 (***跳到*** **NONHOS**) / **99**拒绝回答

**如果选不要确信没有去过任何一家医院,,甚至仅仅去看门诊，体检或拿药.**

**可问：“确实没去过吗？看门诊，体检都算”**

|  |  |
| --- | --- |

AD90

**AD90: 在过去的90天中, 你一共有多少次住院治疗，或者在观察室，急诊室，和抢救室治疗24小时或以上的?**

填写你住院的次数. **00**=没有/ **88**=不知道 / **99**拒绝回答

**EW90: 在过去的90天中, 你还有多少次在观察室，急诊室或者抢救室治疗，但是每次时间不到24小时?**

|  |  |
| --- | --- |

EW90

填写次数. **00**=没有/ **88**=不知道 / **99**拒绝回答

**OP90:最后, 在过去的90天中, 你*还*去过医院看过多少次病，但只是门诊治疗(不包括急诊)?**

|  |  |
| --- | --- |

OP90

填写次数. **00**=没有/ **88**=不知道 / **99**拒绝回答

|  |  |
| --- | --- |

AD12

**AD12 除了上面已经提到的在90天之内的住院，在观察室，急诊室或者抢救室治疗，在过去的*一年*中，**

**你还有多少次住院治疗或者在观察室，急诊室或者抢救室治疗超过一夜？**

填写次数. **00**=没有/ **88**=不知道 / **99**拒绝回答

(如果病人前面回答有过24小时或以上的住院，)**现在我想问你过去1年最近的一次住院，进观察室，急诊室或者抢救室超过24小时的医疗花费情况**

(如果病人前面回答有过24小时以下的住院，)问**现在我想问你过去1年最近的一次进观察室的情况急诊室或者抢救室不到24小时的医疗花费情况**

(如果病人前面回答看过**门诊**，)问**现在我想问你过去1年最近的一次看门诊的医疗花费情况**

| **医院使用** | **最近超过24小时入院** | **最近少于24小时入院** | **最近门诊就诊** |
| --- | --- | --- | --- |
| **最近一次住院，住观察室，急诊室，或抢救室超过24小时的医院的名称** 如果没有入院过夜, 填写 00 | HNAD | | |
| **最近一次住院，住观察室，急诊室，或抢救室少于24小时的医院的名称** 如果没有入院过夜, 填写 00 | **HNOP** | | |
| **最近一次看门诊的医院的名称** 如果没有入院过夜, 填写 00 | **HNEW** | | |
| **你能记起大概的就诊日期吗?** YYMMDD（年月日）  记不起来填99 | **ADDT** | **EWDT** | **OPDT** |
| **医院所有权?**  **1.** 公立 / **2.** 私立(盈利)/ **3.** 私立(非盈利) / **4**其他 /**88**不知道/ **99** 拒绝回答 | **TYAD** | **TYEW** | **TYOP** |
| **医院种类?**  **1.** 中医 / **2.** 西医/  **3.** 中西医结合/ **4**其他/**88**不知道/ **99** 拒绝回答 | **WTAD** | **WTEW** | **WTAD** |
| **医院的等级?**  **1**三级 / **2**二级/ **3**一级 / **4**社区或乡镇/ **5** 其他 | **LVAD** | **LVEW** | **LVOP** |
| **这次就诊的原因是?**  住院的最主要的原因: 心脏病=**1**, 中风=**2**, 肾脏病=**3**, 眼病=**4**, 腿或脚的溃疡=**5**, 癌症=**6**, 肺脏疾病=**7**, 外伤=**8**, 糖尿病=**9**, 分娩=**10**, 消化系统疾病=**11**， 其他=**66**, 不知道=**88** 拒绝回答=**99**  如果是心脏病，是那种心脏病？**1** 心肌梗塞 (/**2** 心绞痛/**3** 中风  **/4**冠心病/ **5** 其他 (请注明) /**88**不知道/ **99** 拒绝回答 | **P1AD**  **P1ADS** | **P1EW**  **P1EWS** | **P1OP**  **P1OPS** |
| 这次住院(就诊)的次主要的原因: 编码与上行相同， **00**=没有  如果是心脏病，是那种心脏病？**1** 心肌梗塞/**2** 心绞痛 /**3** 中风 **/4**冠心病/ **5** 其他(请注明)  /**88**不知道/ **99** 拒绝回答 | **P2AD**  **P2ADS** | **P2EW**  **P2EWS** | **P2OP**  **P2OPS** |
| 这次住院(就诊)的第三主要的原因：编码与上行相同， **00**=没有  如果是心脏病，是那种心脏病？**1** 心肌梗塞/**2** 心绞痛 /**3** 中风 **/4**冠心病/ **5** 其他(请注明)  /**88**不知道/ **99** 拒绝回答 | **P3AD**  **P3ADS** | **P3EW**  **P3EWS** | **P3OP**  **P3OPS** |
| **在医院里你度过了几个晚上/几个小时？**  从住院开始 /**99**-记不清了 如果1小时30分钟，记为1.5小时 | **NIAD** | **DAEW** | **DAOP** |
| **你在病房住了几晚?** | **NIWA** |  |  |
| **你在重症监护病房(ICU)住了几晚** 核对病房的夜晚 + ICU 夜晚 = 总的夜晚. | **NICU** |  |  |
| **住院：内科还是外科病房(观察室，急诊室或抢救室) ？**  **门诊：看过几个(次) 专家门诊?**  **00**=没有去看任何医生 /**88** 不知道/**99** 不知道 | **SPAD** | **SPEW** | **SPOP** |
| **看过几个(次) 普通门诊?**  **00**=没有去看任何医生 /**88** 不知道/**99** 不知道 | **PCAD** | **PCEW** | **PCOP** |
| **做了哪些化验检查或者检查操作?** 将发生的所有代码填全.  **1** 验血 / **2** 验尿 / **3** x线或者B超 / **4**住院手术/ **5**门诊手术/**6** 心电图(ECG) /**7** CT/ **8** 激光治疗/ **9**截肢 **/10**肾脏透析/ **66** 其他/ **88**=不知道 /**99**=没有 | **TSAD** | **TSEW** | **TSOP** |
| **是吃药接受口服药物，打针肌注，还是输液静脉点滴治疗?**  将发生的所有代码填全. **1** 吃药药物 / **2**打针或者肌注 / **3** 输液静脉滴注 / **66** 其他 | **MEAD** | **MEEW** | **MEOP** |
| **这次去这家医院路上花了多长时间(小时)?**  填写小数, 例如, 1.5 = 1小时30分 /**88**不知道 / **99**拒绝回答 | **TRAD** | **TREW** | **TROP** |
| **你怎么去医院?** **1** 步行 / **2** 自行车 / **3** 摩托车 / **4** 公交车 / **5** 私人汽车 / **6** 出租车 / **7** 救护车/**8**火车(外地)/**9**飞机 (外地)/ **66** 其他 / **88** 不知道/**99** 拒绝回答 | **MOAD** | **MOEW** | **MOOP** |
| **途中有多少人陪同你一起来?**  填写人数, 0 如果只有患者. | **PEAD** | **PEEW** | **PEOP** |
| **来医院的总的单程路费是多少(包括陪同人员)?**  填写单程费用，以元为单位 | **TCAD** | **TCEW** | **TCOP** |
| **在住院过程中你和你的家人有多少没有收据的非正式花费(例如红包，请护工)?**  以整元为单位 **777777**-记不清了 | **PYAD** | **PYEW** | **PYPC** |
| **这次住院陪伴你的人住宿，吃饭花了多少钱?**  以整元为单位 **777777**-记不清了 | **PYADB** | **PYEWB** | **PYPCB** |
| **在这次就诊过程中，你和你的家人的所有的花费?** (包括药费, 检验费, 食物, 门诊, 给医生的红包和化验费, 除外路费)  以整元为单位 **777777**-记不清了 | **TPYAD** | **TYEW** | **TYOP** |

**NONHOS** 读: **现在我要询问你一些非医院就诊的一些医疗情况. 同样, 我只想知道从[提到主要事件]开始，最近90天你得到的医疗服务。 在过去的90天, 除了上医院看病之外(包括医院开的诊所) ，你还去其他地方看过病吗？**

**念非医院就诊的种类，如果有，根据表格选项继续询问那一或多类的医疗服务使用情况。**

**1** 是 (填下表)/ **2**.不 (***转至* NCD**)

**88=不知道(*转至* NCD)/ 99拒绝回答**

| 非医院就诊 | **看过多少次?**  (过去的90天内) | **类型** (1 公立 / 2 私立(盈利)/ 3私立(慈善或者非盈利)** | **路程** (单程, 最近一次就诊，以小时来计算，填写小数, 例如, 1.5 = 1小时30分) | **路费**  (单程, 最近一次就诊) | **总体费用***  (最近一次就诊) | **非自付费用(例如保险，单位报销)** |
| --- | --- | --- | --- | --- | --- | --- |
| **西医诊所 (不包括属于医院的)** | **WM90** | **WMTY** | **WMTT** | **WMTC** | **WMCO** | **WMOOP** |
| **中医诊所(不包括属于医院的)** | **TM90** | **TMTY** | **TMTT** | **TMTC** | **TMCO** | **TMOOP** |
| **民间医生(包括草医,江湖医生，气功师，神汉等)** | **TH90** | **THTY** | **THTT** | **THTC** | **THCO** | **MAOOP** |
| **药剂师或者药店** | **PH90** | **PHTY** | **PHTT** | **PHTC** | **PHCO** | **PHOOP** |
| **社区保健工作者** | **CH90** | **CHTY** | **CHTT** | **CHTC** | **CHCO** | **CHOOP** |

*总体费用指病人所接受的医疗服务的全部费用，包括就诊之后的费用，所有药物，辅助材料，操作和检验费用(不管是否能够报销)

**INF:现在我要问你过去90天之内你曾有过的暂时性的疾病或状况. 从[提到的主要事件] ， 你有下列哪些…**

**1** 是 / **2** 不 / **88** 不知道 **99**拒绝回答

| **过性的病征** | **回答** |
| --- | --- |
| 活动性结核 | TD1 |
| 急性疟疾发作 | TD2 |
| 流行性感冒 (Flu) | TD3 |
| 肺炎 | TD4 |
| 腹泻3天或3天以上或者需要药物，输液 | TD5 |
| 怀孕或分娩 | TD6 |
| 其他主要的感染 | TD7 |
| 其他主要的寄生虫 | TD8 |
| 外伤 | TD9 |
| “感冒” 或其他病毒 | TD10 |
| 伤寒 | TD11 |
| 霍乱 | TD12 |
| 其他暂时性疾病 | TD13 |

**NCD: 请告诉我有没有医生说你得过下列疾病(可多选).**

**1** 是 / **2** 不 / **88** 不知道 **99**拒绝回答

| **慢性病征** | **回答** |
| --- | --- |
| 急性心脏病 | NCD1 |
| 心力衰竭 | NCD2 |
| 其他心脏疾病 | NCD3 |
| 中风 | NCD4 |
| 高血压 | NCD5 |
| 癌症 | NCD6 |
| 哮喘 | NCD7 |
| 其他肺部疾病 | NCD88 |
| HIV/AIDS | NCD99 |
| 勃起功能障碍或性欲减退 | NCD10 |
| 肾脏疾病 | NCD11 |
| 截肢(包括脚趾，脚或下肢) | NCD12 |
| 眼部手术 | NCD13 |
| 周围神经病变 (脚或趾的疼痛, 麻刺, 或者麻木) | NCD14 |
| 足或腿的溃疡 (不能自己愈合的溃疡) | NCD15 |
| 眼睛激光治疗 | NCD16 |
| 肾脏透析 | NCD17 |
| 抑郁症 | NCD18 |
| 其他精神疾病 | NCD19 |
| 其他不可治愈的慢性病征 | NCD20 |
| 慢性肝炎added item: chronic hepatitis | NCD21 |
| 慢性消化系统疾病 added item: chronic digestive system diseases | NCD22 |

**TST:** **在过去的90天 (3个月), 你有没有进行过下列化验或者检查? 自己做的不要包括在内**

**1** 是 / **2** 不 / **88** 不知道/ **99** 拒绝回答**.**

| **糖尿病相关检测** | **回答** |
| --- | --- |
| 任何尿检 | TST1 |
| 静脉抽血检查 | TST2 |
| 手指针刺血液检查 | TST3 |
| 血压测量 (手臂袖带) | TST4 |
| 眼睛检查(视力检查除外) | TST5 |
| 足部感觉检查 | TST6 |
| 量体重 | TST7 |
| 用专用卷尺测量腰围 | TST8 |

| **健康问题的影响** |
| --- |

**现在我想知道你的疾病和其他健康方面的问题对你和你家人的生活影响.**

**由于你的疾病和健康方面的问题，你和你的家人生活是否受到影响？**

如果回答“是”，**是谁**(你自己或那个家人，根据病人情况，年龄，可给出家庭成员的种类

**1** 是 / **2**不 / **88** 不适用/不知道/ **99** 拒绝回答

对家庭成员的问题: **1** 配偶 2母亲 / **3** 父亲 / **4** 兄弟 姐妹 / **5** 女儿 儿子 / **6** 孙女， 孙子 / **7** 其他 (要记录3个人)

在这个表格, 家庭成员不限于与应答者一起生活的人，还包括有血缘关系或领养的亲属.

**调查员注意：有些问题只有应答者才回答，有些问题只有家庭成员才回答**

|  | **应答者** | **家庭成员** | **家庭成员** | **家庭成员** |
| --- | --- | --- | --- | --- |
| 使**你自己**不能从事任何能够获得收入的工作 | IR1 |  |  |  |
| 使**你自己**不得不降低工作时间(只包括可以取得收入的工作)？ | IR2 |  |  |  |
| 使**你自己**不得不做更多的工作以增加收入? | IR3 |  |  |  |
| 造成你的部分**家庭成员**不能从事任何能够获得收入的工作? |  | IF1A | IF1B | IF1C |
| 造成你的部分**家庭成员**不得不做更多的工作(只包括可以取得收入的工作)? |  | IF2A | IF2B | IF2C |
| 使你的部分**家庭成员**不得不做更多的工作以增加收入？ |  | IF3A | IF3B | IF3C |
| 使**你自己**不能做任何农活或家务劳动? | IR5 |  |  |  |
| 造成你的其他**家庭成员**不能做任何农活或家务劳动? |  | IF5A | IF5B | IF5C |
| 使你**自己不**去上学或者参加培训? | IR6 |  |  |  |
| 使得其他**家庭成员**不能上学或参加培训? |  | IF6A | IF6B | IF6C |
| 使**你自己**不能去走亲访友? | IR7 |  |  |  |
| 使**你自己**不能得到足够的食物? | IR8 |  |  |  |
| 造成你的其他任何**家庭成员**不能得到足够的食物? |  | IF8A | IF8B | IF8C |

**MISS:** **在过去的90天, 由于健康原因，你有多少天不能进行日常活动(工作, 家务 或学习)?**

|__|__|__| 天 MISS

**88**/不知道 /**99**拒绝回答

**HIRE: 由于你的健康问题，你或者你的家庭是否雇佣其他人来照顾你?**

HIRE

**1** 是 / **2** 不 / **88** 不记得 / **99** 拒绝回答

|__|__|__|__|__|__|__|__|__|__| PAY

**PAY: 如果是的话，每个月要付这个人多少钱**(以整元为单位**)?**

**PAYA: 包吃住吗?**

PAYA

**1** 是 / **2** 不 / **88** 不记得 / **99** 拒绝回答

FCG

**FCG: 由于你的健康问题，是否有你的家庭成员在照顾你?**  **1** 是 / **2** 不/ **88** 不记得/ **99** 拒绝回答

小时 FCGT1

**FCGT: 如果回答¨是¨的话，这个人一天花几个小时来照顾你?**

**调查员换算成百分比 (按24小时计算)**

FCGT

**1**: 1 到 25% / **2**: 26 到 50% / **3**: 51 到 75% / **4** 76 到 100%

**BTA: 在过去的12个月,你的医疗费用和家庭护理费用有那些途径来支付(包括医疗卫生服务，药物，医疗器械和物资)?**

**1** 是 / **2** 不 / **88** 不知道 99拒绝回答

| **经费的种类** | **回答** |
| --- | --- |
| 医疗保险 | BTA10 |
| 花费目前家庭的收入 | BTA1 |
| 社会福利支持 | BTA2 |
| 由单位或其他机构捐赠药品或其他医疗物资 | BTA3 |
| 亲戚或朋友资助(不需要还) | BTA4A |
| 家庭储蓄，例如银行存款 | BTA6 |
| 向别人借款 | BTA7 |
| 变卖家庭财产 (不包括房屋) | BTA8 |
| 变卖房屋 | BTA 9 |

**ACCA: 你有没有生病了不能去看病的情况？**

**1** 是/ **2** 不/ **88** 不知道/ **99**拒绝回答

**ACCB: 请告诉我在过去的12个月中，下列那些原因使你得不到医疗服务和药品:** **1** 是 / **2** 不 / **88** 不知道/ **99**拒绝回答

| **原因** | **回答** |
| --- | --- |
| 没有足够的钱支付 | ACCB1 |
| 交通不便利 | ACCB2 |
| 没有人帮助我到达医疗机构所在地 | ACCB3 |
| 路途太远 | ACCB4 |
| 等的时间太长 | ACCB5 |
| 没有医疗保险 | ACCB6 |
| 病情太重不能前往 | ACCB7 |
| 没有医生或其他医护人员 | ACCB8 |
| 没有药物 | ACCB9 |
| 不相信治疗有用 | ACCB10 |
| 不知道去哪里 | ACCB11 |
| 其他一些原因 | ACCB12 |

医疗保险问题

INS1

INS1 **在过去的一年中，你有没有医疗保险？**

**1** 是 (***转到go to*** *INS3)*, / **2** 不/ **88** 不知道99拒绝回答

INS2 **如果没有，为什么没有？**

**1** 是/ **2** 不/ **88** 不知道99拒绝回答**回答后*转到* *SAT1***

| 原因 | 回答 |
| --- | --- |
| 太贵了，买不起 | INS21 |
| 不需要 | INS22 |
| 没有地方买 | INS23 |
| 不知道如何买 | INS24 |
| 其他一些原因 | INS25 |

INS3：**如果你有医疗保险，你目前参加的社会(非商业) 医疗保险是**?

**1** 是/ **2** 不/ **88** 不知道/ 99拒绝回答

| 原因 | 回答 |
| --- | --- |
| 城镇基本医疗保险 | INS31 |
| 大病医疗保险 | INS32 |
| 公费医疗 | INS33 |
| 劳保医疗 | INS34 |
| 合作医疗 | INS35 |
| 其他社会医疗保险 | INS36 |

INS4a **如果你有医疗保险，你的门诊医药费用负担形式是那种？** (多重选择)

INS4a

**1** 按比例报销制/ **2** 月或年费用包干制 (***跳转至go to* INS4c**) / **3** 其他形式/ **88**不知道/ **99**拒绝回答

INS4b **如果是报销制或其他形式，你自己需要负担门诊医药费用的比例**? (大概估计)

%

INSb

**88**不知道/ **99**拒绝回答

INS4c **如果是费用包干制，每年平均发放给你的包干费用为** (大概估计) ____元/**年**

INS4c

如病人回答 ___元/月换算成以年为单位

**88**不知道/ **99**拒绝回答

INS4d **如果你住院, 你自己需要负担住院费用的比例** (大概估计)

_____%

INS4d

**88**不知道/ **99**拒绝回答

INS5

INS5 **你有没有购买商业医疗保险？**

**1**有/ **2**没有 / **88**不知道/ **99**拒绝回答

**看病就诊的经历

SAT1你对最近看病就诊的经历满意吗？**

**(如果病人1年内未看过病，可不回答，填00)**

**1** 满意(***跳转至SMOK***) / **2** 不满意/ **3**无所谓 (***跳转至SMOK***) / **88**不知道 (***跳转至SMOK***) / **99**拒绝回答 (***跳转至SMOK***)

**SAT2 如果你不满意，为什么？**

**1** 是/ **2** 不/ **88** 不知道/**99**拒绝回答

| 原因 | 回答 |
| --- | --- |
| 服务态度差 | SAT21 |
| 技术水平低 | SAT22 |
| 设备环境差 | SAT23 |
| 提供不必要的服务(药物，检查) | SAT24 |
| 收费不合理 | SAT25 |
| 医疗费用高 | SAT26 |
| 不能赊帐 | SAT27 |
| 看病手续繁琐 | SAT288 |
| 等候时间过长 | SAT299 |
| 到医院不方便 | SAT210 |
| 其他 | SAT211 |

SMOK

**SMOK:** **现在你是否吸烟( 包括香烟，烟斗，旱烟，雪茄，嚼烟)?**

**1** 是 (**转到 go to SMKQ**)/ **2** 不/ **99** 拒绝回答

SMKP

**SMKP: 如果回答”不”，你过去是否吸过烟?**

**1** 曾经吸烟/ **2** 从不吸烟 (**转到go to ASPN**) / **99** 拒绝回答或不确信

**SMKQ：如果你现在吸烟，你是每天吸还是有些时候吸？**

SMKP

**1**每天吸/**2**有时候吸/ **88**不知道/**99**拒绝回答

**SMKS:** **如果你现在吸烟，是否有医务人员建议你戒烟呢?**

SMKS

**1**是(建议戒烟]/ **2** 否/ **99** 拒绝或不确信

| **药物治疗** |
| --- |

ASPN

**ASPN**: **每天都服用阿司匹林吗?**

**1** 是/ **2** 不/**88** 不知道 **99** 拒绝回答

MED

**MED:** **你现在有没有服用，注射，或使用其他的药(包括外用药，喷雾剂，或为慢性病急性发作备用的药物)**?

**1** 是/ **2** 不/ **88** 不知道 **99** 拒绝回答

**如果是的话，我能看一看你用的药并且问一些问题吗?**

让应答者出示他或她最近吃的所有药片,胰岛素,以及其他药物. 每一种填完下表.

| 药名(如为胰岛素，胰岛素注射器，注射笔，请记下品牌 | 每天每片/只针剂/喷药剂量(以毫克为单位)或/及每天每次胰导素剂量(如每次剂量不同，请分别列出) | 每天应该服用药片数/注射胰导素次数 | 实际每天服用药片数/注射胰导素次数(如果使用的话) | 服药/注射频率(以每周计算)1. 每天2. 4-6 天/周3. 1-3 天/周4. < 1天/周 | **是否按照处方服药，如果没有，主要原因是**  0 按处方服用  1 副作用  2 不能负担费用  3 忘记  4 药难以取得  5 没有人帮助服药  6 注射器械的问题  7不相信有效  8 不需要  88 不知道  99拒绝回答 | 在哪里取得药物?1. 药店2. 医院或诊所药房3. 药摊 4. 亲友  5. 其他 99. 拒绝回答refused | 最近一次买药总共花费了你多少钱(包括可以报销的部分)? | 得到了多少药片/多少剂胰导素? |
| --- | --- | --- | --- | --- | --- | --- | --- | --- |
| M1A | M1B | M1C | M1D | M1E | M1F | M1G | M1H | M1I |
| M2A | M2B | M2C | M2D | M2E | M2F | M2G | M2H | M2I |
| M3A | M3B | M3C | M3D | M3E | M3F | M3G | M3H | M3I |
| M4A | M4B | M4C | M4D | M4E | M4F | M4G | M4H | M4I |
| M5A | M5B | M5C | M5D | M5E | M5F | M5G | M5H | M5I |
| M6A | M6B | M6C | M6D | M6E | M6F | M6G | M6H | M6I |
| M7A | M7B | M7C | M7D | M7E | M7F | M7G | M7H | M7I |
| M88A | M88B | M88C | M88D | M88E | M88F | M88G | M88H | M88I |
| M99A | M99B | M99C | M99D | M99E | M99F | M99G | M99H | M99I |

**如果对照组，转至ETIM。**

(如果IGR 病人用药或测血糖，也要继续问)

**对糖尿病病人的问题**

|__|__| 年

DM2

**DM2:** **你得糖尿病几年了?**)

. **88** 不知道 / **99**拒绝回答

DM21

**DM21： 你现在使用胰岛素吗？**

**1** 是/**2** 否/ **88** 不知道/ **99**拒绝回答

DM22

**DM22 你现在使用口服降糖药吗？**

**1** 是/ **2** 否/ **88** 不知道/ **99**拒绝回答

**DM3:** **在过去的90天，你自己多久测一次血糖(包括家人，朋友，家庭护理人员为你测的，但不包括医护人员为你测的)?**

**每天__________次**

DM3d

DM3w

每周__________次

DM3m

每月__________次

**000** 从不

**888** 不知道

**999** 拒绝回答

DM3A

**DM3A 如果你自己测血糖，你上次买了多少血糖测试纸?**

**666**不在家测 **888** 不知道/**999** 拒绝回答

|  |  |  |  |  |
| --- | --- | --- | --- | --- |

DM3B

**DM3B:** **买这些血糖测试试纸花费了你多少钱(元)?**

**6666**不在家测 **8888** 不知道/不适用 /**9999** 拒绝回答

DM3C

**DM3C:** 你**这些试纸从哪里买的?**

**1** 私人医生或者诊所 / **2** 国营医院，诊所 **/ 3** 私人药房 / **4** 其他 / **88**不知道 /**99** 拒绝回答

DM3D

**DM3D:** **在过去的一年，你是否能够得到你需要的所有血糖测试试纸?**

**1** 是的/ **2** 大部分时候可以**/ 3** 经常不行/ **4** 从不 /**88**不知道 /**99** 拒绝回答

**DM4:** **在过去的90天, 医护人员为你进行血糖测试的频率是多少?**

**每天__________次**

DM41

DM42

每周__________次

DM43

每月__________次

**000** 从不

**888** 不知道

**999** 拒绝回答

每次花费: |__|__|__|__|__|__| DM5

**DM5: 医护人员给你测试一次血糖你要付多少钱(取整到元)?**

**888** 不知道 / **999** 拒绝回答

DM6

**DM6：过去90天你是否曾经自己或到医院，诊所注射胰岛素？**

1自己注射(**跳转**至**DM-6H**) /2在诊所或医院注射(**跳转至go to DM-6H**) / **3**没有 (**跳转**至**DM-6A**) /**88**不知道 /**99**拒绝回答

**DM6A: 不注射胰岛素的原因**

**1**是 / **2**不/ **88**不知道 / **99**拒绝回答

| 原因 | 回答 |
| --- | --- |
| 医生没有建议打胰岛素 | DM6A1 |
| 太贵买不起 | DM6A1 |
| 买不到胰岛素 | DM6A2 |
| 胰岛素疗效不佳，有时血糖太高，有时血糖太低 | DM6A3 |
| 药房诊所无针头或注射器 | DM6A4 |
| 针头或注射器太贵负担不起 | DM6A5 |
| 害怕或不喜欢打针 | DM6A6 |
| 不知道如何注射胰岛素 | DM6A7 |
| 不知道什么时候注射胰岛素 | DM6A8 |
| 医生建议打胰岛素但我自己认为我不需要胰岛素治疗 | DM6A9 |
| 找不到或者买不起测血糖的设备 | DM6A10 |
| 找不到医生或护士 | DM6A11 |
| 其他一些原因 | DM6A12 |
| 不知道 | DM6A13 |
| 拒绝回答 | DM6A14 |

DM6H

**DM6H：在过去的90天，有多少天你没有注射胰岛素，其原因是因为没有可用的针头或注射器？**

**1** 0天 /**2** 少于5天 /**3** 6-10天 /**4** 11-20天 /**5** 21 到45天 /**6** 超过半数时间 /**7** 总是(因为没有注射器过去90天都没有打胰岛素/

**88** 不知道/ **99** 拒绝回答

DM6I

**DM6I：在过去的90天，有多少次你给自己注射胰岛素时重复使用针头或注射器？**

**1**从来没有(每次总是用新的) /**2** 2次 /**3** 3次 /**4** 4次 /**5** 5次或以上, 但不总是/ **6**总是

**88** 不知道/ **99** 拒绝回答

**DM7 你听说过有关糖尿病自我控制的信息吗？**

**1**听说过/ **2**没有听说过/ **88**不知道/ **99** 拒绝回答

**DM7A 如果听说过，信息从那儿来？(可多选)**

**1是/ 2不/ 88不知道/ 99拒绝回答**

| 种类 | 回答 |
| --- | --- |
| 书籍/报刊杂志 | DM7A1 |
| 媒体/网络 | DM7A2 |
| 医护人员 | DM7A3 |
| 宣传栏 | DM7A4 |
| 其他途径 | DM7A5 |

DM8在过去的一年中，有医生对你说过控制血糖的重要性吗？ In the last

DM8

**1**是/ **2**没有/ **88**不知道/ **99**拒绝回答

DM9

DM9 在过去的一年中，有医生给你查过糖尿病眼底吗？

**1**是/ **2**没有/ **88**不知道/ **99**拒绝回答

DM10

DM10 在过去的一年中，有医生给你查血肌肝或尿蛋白吗?

**1**是/ **2**没有/ **88**不知道/ **99**拒绝回答

DM11

DM11 在过去的一年中，医生有没有检查你的脚底反应?

**1**是/ **2**没有/ **88**不知道/ **99**拒绝回答

**ETIM: 结束时间:**

| **|__|__|** | |__|__| |
| --- | --- |

小时 分钟 ETIM

24 小时制
