# Supplementary material for: Medical Care and Payment for Diabetes in China: Enormous Threat and Great Opportunity
Source: PLoS One. 2012 Sep 26;7(9):e39513. doi: 10.1371/journal.pone.0039513 (PMC3458850; doi:10.1371/journal.pone.0039513)
Supplement: File S2 — Supplementary Tables. Table S2–1. Characteristics of Subjects by Study Site. Table S2.2. Median Use of Inpatient and Outpatient Services. Table S2–3. Mean annual payments (CNY) for “Western” medicines, by where purchased. Table S2–4. Detail of mean annual payments (CNY) for medicines by source, location, and DM/NGT status. Table S2–5. Reason for admission to hospital among subjects who reported an admission during the previous year. Table S2–6. Reason for visits to hospital outpatient clinics among subjects who reported a visit during the previous 90 days. Table S2–7. Results of multivariable hurdle models to test for DM vs. NGT differences in the use of overnight hospital admissions, outpatient visits, and medicines. (DOC) [file pone.0039513.s002.doc]

Supporting Information 2

Supplementary Tables to

Yang W et al. (2012) Medical care and payment for diabetes in china: enormous threat and great opportunity. PLoS ONE.

Table S2-1. Characteristics of Subjects by Study Site

| **Study Site** |  | **N** | **Mean Age** | **s. d. of Age** | **Percent Women** | **Percent Urban** |
| --- | --- | --- | --- | --- | --- | --- |
| **Beijing** | *Diabetes* | *443* | 59.51 | 11.24 | 62.22% | 64.77% |
| **(4 sites)** | *NGT** | *447* | 52.39 | 13.58 | 64.35% | 70.56% |
|  | Total | 890 | 55.86 | 12.98 | 63.29% | 67.68% |
| **Liaoning** | *Diabetes* | *149* | 54.60 | 11.60 | 59.06% | 81.88% |
|  | *NGT** | *146* | 50.52 | 11.62 | 69.18% | 100.00% |
|  | Total | 295 | 52.58 | 11.77 | 64.07% | 90.85% |
| **Shandong** | *Diabetes* | *127* | 54.55 | 13.20 | 48.03% | 87.40% |
|  | *NGT** | *106* | 41.54 | 16.20 | 60.95% | 64.08% |
|  | Total | 233 | 48.66 | 15.98 | 53.88% | 76.96% |
| **Taiyuan,** | *Diabetes* | *151* | 53.72 | 11.26 | 61.59% | 74.17% |
| **Shaanxi** | *NGT** | *142* | 48.51 | 10.91 | 64.79% | 51.41% |
|  | Total | 293 | 51.19 | 11.38 | 63.14% | 63.14% |
| **Shanghai** | *Diabetes* | *176* | 56.48 | 9.72 | 57.39% | 71.43% |
|  | *NGT** | *174* | 55.00 | 9.55 | 53.44% | 66.67% |
|  | Total | 350 | 55.57 | 9.67 | 55.43% | 69.05% |
| **Hunan** | *Diabetes* | *56* | 57.39 | 11.21 | 58.92% | 76.79% |
|  | *NGT** | *157* | 45.43 | 14.76 | 50.96% | 72.44% |
|  | Total | 213 | 48.76 | 14.85 | 53.05% | 73.58% |
| **Fujian** | *Diabetes* | *84* | 58.49 | 11.57 | 51.19% | 92.86% |
|  | *NGT** | *57* | 50.18 | 12.19 | 66.67% | 94.74% |
|  | Total | 141 | 55.16 | 12.46 | 57.45% | 93.62% |
| **Sichuan** | *Diabetes* | *136* | 58.31 | 10.75 | 61.76% | 90.44% |
|  | *NGT** | *139* | 54.75 | 13.17 | 65.23% | 89.86% |
|  | Total | 275 | 56.50 | 12.15 | 63.50% | 90.15% |
| **Xi'an,** | *Diabetes* | *82* | 58.95 | 10.91 | 59.76% | 55.56% |
| **Shaanxi** | *NGT** | *105* | 51.44 | 13.71 | 65.71% | 64.76% |
|  | Total | 187 | 54.73 | 13.07 | 63.10% | 60.75% |
| **Xinjiang** | *Diabetes* | *78* | 60.92 | 8.89 | 47.44% | 100.00% |
|  | *NGT** | *80* | 53.15 | 8.00 | 76.25% | 100.00% |
|  | Total | 158 | 56.99 | 9.28 | 62.03% | 100.00% |
| **All Sites** | *Diabetes* | *1482* | 57.39 | 13.25 | 58.34% | 75.96% |
|  | *NGT** | *1553* | 51.00 | 13.25 | 62.90% | 75.64% |
|  | Total | 3035 | 54.02 | 12.78 | 60.67% | 75.78% |

Table S2-2. Median Use of Inpatient and Outpatient Services

|  | Location | | Age in Years | | | | | Years Since Diabetes Diagnosis | | | | All Subjects |
| --- | --- | --- | --- | --- | --- | --- | --- | --- | --- | --- | --- | --- |
|  | Urban | Rural | < 40 | 40-49 | 50-59 | 60-69 | 70+ | < 2 | 3 to 5 | 6 to 10 | >10 | Total |
| N (DM) | 1121 | 355 | 88 | 259 | 459 | 398 | 246 | 527 | 325 | 280 | 146 | 1481 |
| N (NGT) | 1154 | 392 | 322 | 344 | 445 | 283 | 133 |  |  |  |  | 1553 |
| INPATIENT CARE | |  |  |  |  |  |  |  |  |  |  |  |
| Median Length of Stay, Most Recent Hospital Admission, if Admitted | | | | | | |  |  |  |  |  |  |
| DM | 10 | 11 | 8 | 12 | 10 | 10 | 10 | 10 | 10 | 15 | 9 | 10 |
| nonDM | 10 | 13 | 9 | 10 | 10 | 13 | 7 |  |  |  |  | 12 |
| Ratio | 1.00 | 0.81 | 0.94 | 1.20 | 0.95 | 0.80 | 1.43 |  |  |  |  | 0.87 |
| Median Point of Service Expenditures per Admission (CNY), Most Recent Hospital Admission, if Admitted | | | | | | | | | | |  |  |
| DM | 5800 | 3000 | 3200 | 7000 | 5000 | 13 | 12 | 5250 | 4000 | 5000 | 5000 | 5000 |
| nonDM | 4050 | 6800 | 11867 | 3000 | 3688 | 4000 | 5000 |  |  |  |  | 5000 |
| Ratio | 1.43 | 0.44 | 0.27 | 2.33 | 1.36 | 4500.00 | 4100.00 |  |  |  |  | 1.00 |
| OUTPATIENT VISITS | |  |  |  |  |  |  |  |  |  |  |  |
| Median Point of Service Hospital Outpatient Expenditures per Visit, Most Recent Visit, if Visited (CNY) | | | | | | | | | | |  |  |
| DM | 180 | 200 | 110 | 200 | 200 | 168 | 220 | 153 | 150 | 200 | 280 | 200 |
| nonDM | 150 | 200 | 50 | 135 | 200 | 200 | 200 |  |  |  |  | 170 |
| Ratio | 1.20 | 1.00 | 2.20 | 1.48 | 1.00 | 0.84 | 1.10 |  |  |  |  | 1.18 |
| Median Hospital Outpatient Visits, if Visited (CNY) | | | | | |  |  |  |  |  |  |  |
| DM | 3 | 2 | 1 | 3 | 3 | 3 | 3 | 3 | 3 | 3 | 4 | 3 |
| nonDM | 2 | 2 | 1 | 1 | 2 | 3 | 2 |  |  |  |  | 2 |
| Ratio | 1.50 | 1.00 | 1.00 | 3.00 | 1.50 | 1.00 | 1.50 |  |  |  |  | 1.50 |

Table S2-3. Mean annual payments (CNY) for ”Western” medicines, by where purchased

|  | Hospital | Private Pharmacy | Other | Total |
| --- | --- | --- | --- | --- |
| Insulin | 4553 | 4140 | 2326 | 4304 |
| Oral Agents | 1627 | 1585 | 1758 | 1618 |
| Other DM-Related | 1288 | 994 | 1005 | 1230 |
| DM | 1310 | 1063 | 1059 | 1269 |
| NGT | 1179 | 742 | 628 | 1047 |
| Ratio | 1.11 | 1.43 | 1.69 | 1.21 |
| Other Non-DM-Related | 1397 | 1959 | 405 | 1548 |
| DM | 1301 | 1331 | 438 | 1296 |
| NGT | 1528 | 2562 | 372 | 1862 |
| Ratio | 0.85 | 0.52 | 1.18 | 0.70 |
| TOTAL | 8865 | 8678 | 5495 | 8701 |
| DM | 8791 | 8118 | 5582 | 8487 |
| NGT | 2707 | 3304 | 1001 | 2909 |
| Difference | 6084 | 4814 | 4581 | 5578 |
| Ratio | 3.25 | 2.46 | 5.58 | 2.92 |

Based on self-reported most recent payment, annualized using the self-reported number of pills or units of insulin used per day and the number of pills or units of insulin most recently purchased, and adjusted for self-reported non-adherence (the proportion of days per week when the medicine was taken as directed). Oral agents are antihyperglycemic medicines other than insulin. DM-related medicines are statins, antihypertensives, and aspirin. The “Other” category within places of purchase includes private medical offices, purchases from informal vendors, and acquisition from friends or family members.

Table S2-4. Detail of mean annual payments (CNY) for medicines by source, location, and DM / NGT status.

|  |  | Public Pharmacies | | Private Pharmacies | |
| --- | --- | --- | --- | --- | --- |
| Drug |  | Urban | Rural | Urban | Rural |
| Metformin | DM | 853 | 1202 | 1419 | 651 |
|  | NGT | NA | NA | NA | NA |
| Sulphonylurea | DM | 1688 | 1256 | 1305 | 747 |
|  | NGT | NA | NA | NA | NA |
| Acarbose | DM | 2622 | 2506 | 5075 | 1582 |
|  | NGT | NA | NA | NA | NA |
| Insulin | DM | 4046 | 9451 | 4814 | 2419 |
|  | NGT | NA | NA | NA | NA |
| Glucose Lowering Agents | DM | 3020 | 2234 | 1962 | 767 |
|  | NGT | NA | NA | NA | NA |
| Lipid Lowering Agents | DM | 2521 | 5697 | 1460 | 1304 |
|  | NGT | 3931 | 1825 | 1752 | 4198 |
| Blood Pressure Drugs | DM | 1160 | 737 | 643 | 322 |
|  | NGT | 1509 | 701 | 531 | 1720 |
| Anti Coagulants | DM | 604 | 449 | 441 | 190 |
|  | NGT | 711 | 741 | 156 | 1187 |
| Other NonDM Related | DM | 1341 | 1124 | 1644 | 466 |
|  | NGT | 1791 | 1224 | 2787 | 2086 |

Table S2-5. Reason for admission to hospital among subjects who reported an admission during the previous year

|  | **Diabetes Cases** | | | | **Normal Glucose Tolerance** | | | |
| --- | --- | --- | --- | --- | --- | --- | --- | --- |
| **Reason For Admission** | **N** | **%** | **Length of Stay** | **Mean Payment** | **N** | **%** | **Length of Stay** | **Mean Payment** |
| **Heart Disease** | 14 | 16.67 | 12.17 | 24100 | 6 | 12.00 | 13.00 | 4744 |
| **Stroke** | 4 | 4.76 | 11.50 | 16667 | 5 | 10.00 | 26.00 | 6860 |
| **Kidney Disease** | 1 | 1.19 | 22.00 | 11000 | 1 | 2.00 | NA | NA |
| **Eye Disease** | 4 | 4.76 | 13.67 | 6131 | 1 | 2.00 | NA | 1700 |
| **Leg or Foot Ulcer** | 12 | 14.29 | 10.71 | 11086 | 14 | 28.00 | 21.71 | 7279 |
| **Cancer** | 1 | 1.19 | 9.00 | 1000 | 0 | 0.00 | NA | NA |
| **Lung Disease** | 1 | 1.19 | 29.00 | 21000 | 5 | 10.00 | 17.00 | 10600 |
| **Trauma** | 1 | 1.19 | NA | NA | 0 | 0.00 | NA | NA |
| **Diabetes** | 27 | 32.14 | 14.64 | 15632 | 0 | 0.00 | NA | NA |
| **Childbirth** | 0 | 0.00 | NA | NA | 1 | 2.00 | 3.00 | NA |
| **Digestive Problems** | 8 | 9.52 | 11.67 | 8088 | 5 | 10.00 | 13.25 | 3825 |
| **Other** | 9 | 10.71 | 9.40 | 8186 | 12 | 24.00 | 9.00 | 5278 |
| **Does Not Know** | 2 | 2.38 | NA | NA | 0 | 0.00 | NA | NA |

Table S2-6. Reason for visits to hospital outpatient clinics among subjects who reported a visit during the previous 90 days

|  | **Diabetes** | | | **Normal Glucose Tolerance** | | |
| --- | --- | --- | --- | --- | --- | --- |
| **Reason For Visit** | **N** | **%** | **Mean Payment** | **N** | **%** | **Mean Payment** |
| **Heart Disease** | 42 | 7.09 | 518 | 27 | 9.54 | 500 |
| **Stroke** | 4 | 0.68 | 240 | 2 | 0.71 | 300 |
| **Kidney Disease** | 4 | 0.68 | 700 | 6 | 2.12 | 300 |
| **Eye Disease** | 9 | 1.52 | 293 | 4 | 1.41 | 200 |
| **Leg or Foot Ulcer** | 72 | 12.16 | 380 | 61 | 21.55 | 523 |
| **Cancer** | 3 | 0.51 | 120 | 1 | 0.35 | 120 |
| **Lung Disease** | 6 | 1.01 | 1000 | 5 | 1.77 | 433 |
| **Trauma** | 4 | 0.68 | 200 | 5 | 1.77 | 310 |
| **Diabetes** | 314 | 53.04 | 301 | 0 | 0.00 | NA |
| **Childbirth** | 0 | 0.00 | NA | 0 | 0.00 | NA |
| **Digestive Problems** | 17 | 2.87 | 200 | 19 | 6.71 | 634 |
| **Other** | 111 | 18.75 | 100 | 151 | 53.36 | 211 |
| **Refused** | 6 | 1.01 | NA | 1 | 0.35 | 50 |
| **Does Not Know** | 0 | 0.00 | NA | 1 | 0.35 | 150 |

Table S2-7. Results of multivariable hurdle models to test for DM vs. NGT differences in the use of overnight hospital admissions, outpatient visits, and medicines.

INPATIENT

Call:

hurdle(formula = hosp.ad ~ case + age + sex + location | case + age +

sex + location, data = y)

Pearson residuals:

Min 1Q Median 3Q Max

-0.3409 -0.1733 -0.1369 -0.1047 32.0341

Count model coefficients (truncated poisson with log link):

Estimate Std. Error z value Pr(>|z|)

(Intercept) -1.496494 1.275474 -1.173 0.241

caseDiabetes 0.263999 0.472098 0.559 0.576

age -0.003174 0.018620 -0.170 0.865

sexMale -0.098783 0.412579 -0.239 0.811

locationUrban 1.049966 0.734709 1.429 0.153

Zero hurdle model coefficients (binomial with logit link):

Estimate Std. Error z value Pr(>|z|)

(Intercept) -6.37934 0.65643 -9.718 < 2e-16 ***

caseDiabetes 0.60044 0.25048 2.397 0.016521 *

age 0.03939 0.01049 3.754 0.000174 ***

sexMale 0.44857 0.23102 1.942 0.052171 .

locationUrban -0.04588 0.27594 -0.166 0.867934

---

Signif. codes: 0 '***' 0.001 '**' 0.01 '*' 0.05 '.' 0.1 ' ' 1

Number of iterations in BFGS optimization: 20

Log-likelihood: -399.3 on 10 Df

OUTPATIENT

Call:

hurdle(formula = tot.op1 ~ case + age + sex + location | case + age +

sex + location, data = y)

Pearson residuals:

Min 1Q Median 3Q Max

-1.1833 -0.5791 -0.4219 -0.0492 17.0619

Count model coefficients (truncated poisson with log link):

Estimate Std. Error z value Pr(>|z|)

(Intercept) -0.072970 0.114809 -0.636 0.525

caseDiabetes 0.224520 0.042656 5.264 1.41e-07 ***

age 0.015619 0.001782 8.765 < 2e-16 ***

sexMale -0.026448 0.037653 -0.702 0.482

locationUrban 0.333232 0.056323 5.916 3.29e-09 ***

Zero hurdle model coefficients (binomial with logit link):

Estimate Std. Error z value Pr(>|z|)

(Intercept) -3.257108 0.217790 -14.955 < 2e-16 ***

caseDiabetes 0.869843 0.086232 10.087 < 2e-16 ***

age 0.029750 0.003594 8.279 < 2e-16 ***

sexMale -0.141875 0.085900 -1.652 0.0986 .

locationUrban 0.491648 0.103664 4.743 2.11e-06 ***

---

Signif. codes: 0 '***' 0.001 '**' 0.01 '*' 0.05 '.' 0.1 ' ' 1

Number of iterations in BFGS optimization: 15

Log-likelihood: -4153 on 10 Df

MEDICINES

## Mean number western meds per person

Call:

hurdle(formula = total.drug ~ case + age + sex + location | case + age +

sex + location, data = y)

Pearson residuals:

Min 1Q Median 3Q Max

-1.4985 -0.5152 -0.3304 0.2938 11.6260

Count model coefficients (truncated poisson with log link):

Estimate Std. Error z value Pr(>|z|)

(Intercept) -1.37213 0.19462 -7.050 1.79e-12 ***

caseDiabetes 0.39431 0.07938 4.967 6.79e-07 ***

age 0.02174 0.00282 7.709 1.27e-14 ***

sexMale 0.05457 0.05798 0.941 0.347

locationUrban 0.07502 0.07354 1.020 0.308

Zero hurdle model coefficients (binomial with logit link):

Estimate Std. Error z value Pr(>|z|)

(Intercept) -4.056105 0.235113 -17.252 < 2e-16 ***

caseDiabetes 2.004558 0.089241 22.462 < 2e-16 ***

age 0.044729 0.003845 11.634 < 2e-16 ***

sexMale -0.150029 0.089667 -1.673 0.09429 .

locationUrban 0.311424 0.103360 3.013 0.00259 **

---

Signif. codes: 0 '***' 0.001 '**' 0.01 '*' 0.05 '.' 0.1 ' ' 1

Number of iterations in BFGS optimization: 12

Log-likelihood: -3123 on 10 Df

INPATIENT WITH CASE ONLY

Call:

hurdle(formula = hosp.ad ~ case | case, data = y)

Pearson residuals:

Min 1Q Median 3Q Max

-0.1762 -0.1762 -0.1183 -0.1183 22.4345

Count model coefficients (truncated poisson with log link):

Estimate Std. Error z value Pr(>|z|)

(Intercept) -0.8056 0.3949 -2.040 0.0414 *

caseDiabetes 0.2133 0.4622 0.461 0.6445

Zero hurdle model coefficients (binomial with logit link):

Estimate Std. Error z value Pr(>|z|)

(Intercept) -4.1122 0.2016 -20.395 < 2e-16 ***

caseDiabetes 0.8220 0.2454 3.349 0.00081 ***

---

Signif. codes: 0 '***' 0.001 '**' 0.01 '*' 0.05 '.' 0.1 ' ' 1

Number of iterations in BFGS optimization: 12

Log-likelihood: -411.7 on 4 Df

TOTAL OUTPATIENT WITH CASE ONLY

Call:

hurdle(formula = tot.op1 ~ case | case, data = y)

Pearson residuals:

Min 1Q Median 3Q Max

-0.7120 -0.7120 -0.4372 0.1398 20.6923

Count model coefficients (truncated poisson with log link):

Estimate Std. Error z value Pr(>|z|)

(Intercept) 1.05372 0.03541 29.76 < 2e-16 ***

caseDiabetes 0.32488 0.04112 7.90 2.79e-15 ***

Zero hurdle model coefficients (binomial with logit link):

Estimate Std. Error z value Pr(>|z|)

(Intercept) -1.36073 0.06296 -21.61 <2e-16 ***

caseDiabetes 1.01012 0.08215 12.30 <2e-16 ***

---

Signif. codes: 0 '***' 0.001 '**' 0.01 '*' 0.05 '.' 0.1 ' ' 1

Number of iterations in BFGS optimization: 11

Log-likelihood: -4358 on 4 Df
