# Supplementary material for: Medical Care and Payment for Diabetes in China: Enormous Threat and Great Opportunity
Source: PLoS One. 2012 Sep 26;7(9):e39513. doi: 10.1371/journal.pone.0039513 (PMC3458850; doi:10.1371/journal.pone.0039513)
Supplement: File S3 — Links to Source Data, Data Entry Program, and Data Analysis Program. (DOC) [file pone.0039513.s003.doc]

Supporting Information 3

Source Datasets, Data Entry Program, and Analytic Programs used to generate:

Yang W et al. (2012) Medical care and payment for diabetes in china: enormous threat and great opportunity. PLoS ONE.

To allow open confirmation of our results and to give open access to our microdata so that others can perform and published further analyses, we have posted our data entry program, source data and programs for data preparation and analysis to the permanent website of the International Diabetes Federation. The link to the webpage where all these resources can be downloaded is <http://www.idf.org/national-study-diabetes-impact-and-care-china>. This annex lists the file names and describes their contents.

**INTERVIEW SCHEDULE**

The interview schedule, itself, is reproduced in Supporting Information 1. This schedule is written in Mandarin. There is no exact copy of the Mandarin version in English. However, the interview schedules used in Africa are very similar, and any question with the same field number (variable name) is exactly identical in the Chinese version. The entire study protocol, including interview schedules for the African studies can be downloaded in English or French from <http://www.idf.org/diabetes-social-and-economic-impact-studies>.

**DATA ENTRY PROGRAM**

The data entry program was written in Epi Info™ to run on personal computers on which the free, public-domain Epi Info™ software had been installed. To download the program, got to <http://www.idf.org/national-study-diabetes-impact-and-care-china>. To download the software, go to <http://wwwn.cdc.gov/epiinfo/html/downloads.htm>. To run the data entry programs used in the IDF Diabetes Impact Studies, download the older Epi Info platform, Epi Info™ 3.5.3, rather than the new Epi Info™ 7.

**SOURCE DATA FILES**

All study datasets are provided as CSV (comma-separated variable) flat files that R can open and that the user also can view in EXCEL or in other spreadsheet and database programs. The Source Data Files are the datasets generated by our Epi Info™ data entry program. Because the interview was long, we divided the data entry program into five sections that created, in turn, five separate source datasets:

CHINA_DEMOGRAPHICS_SUBMITTED_SOURCE.csv

CHINA_HUI_SUBMITTED_SOURCE.csv *(HUI stands for Health Utility Index)*

CHINA_SERVICES_SUBMITTED_SOURCE.csv

CHINA_IMPACT_SUBMITTED_SOURCE.csv

CHINA_MEDS_SUBMITTED_SOURCE.csv

The versions of these datasets that are available to download are not the completely raw data: personal identifiers have been removed, the data have been verified against the hardcopy questionnaires, cleaned (in EXCEL) to remove implausible and inconsistent values, and in a very few instances of service utilization and payment values, winsorized to reduce extreme values to the level of the highest remaining natural value of the variable.

Datasets from the original Chinese national screening study, from which we drew our participants, and against which our subjects are compared in the paper, are not included.

**PROGRAMS FOR DATA CLEANING, TRANSFORMATION, AND ANALYSIS; AND ANALYSIS DATASETS**

The data-cleaning and data-analysis programs were written in R, a free, open source program for statistical computing. We provide text files that R will recognize and run. To download and learn about R, go to <http://www.r-project.org/>

We used the R program, CHINA_DATA_CODING_RCODE.txt, to recode and transform variables into forms more convenient for analysis and to create three amalgamated datasets for use in statistical analyses. The last lines of CHINA_DATA_CODING_RCODE.txt generate output datasets “tot”, “x”, and “y”, which yield, respectively:

CHINA_CLEAN_RECODED_SOURCE.csv, an amalgamation of the five source datasets that was not used in subsequent analyses;

CHINA_PERSON_DATA.csv, a collection of all the original and transformed variables and values at the person-level, i.e., a dataset with one value per variable per person; and

CHINA_MEDS_DATA.csv, a collection of all the data relating to medicines, transformed so that the unit of observation is the person-medicine, i.e., one observation for each medicinal compound reported by each person in the study.

The R program, CHINA_PERSON_ANALYSIS_RECODE.csv, inputs the dataset CHINA_PERSON_DATA.csv and outputs tabulations, regressions, and test results for persons.

The R program, CHINA_MEDICINE_ANALYSIS_RECODE.csv, inputs the dataset CHINA_MEDS_DATA.csv and outputs tabulations and statistical test results for person-usages of medicines.

The outputs of each program were then converted to reports in pdf format, from which the tables in the paper were constructed, using EXCEL.
